# Dietary Regulation of Intestinal Stem Cell Function: Nutrient-Sensing, Microbiota-Mediated, and Regenerative Mechanisms from a Dietetic Perspective

**DOI:** 10.3390/biomedicines14081873

**Published:** 2026-08-21

**Authors:** Elif Akbaş, Beyza Nur Gürgen, Ece Akgül, Esra Günay, Burçak Tok, Ecem Ozduran, Saliha Ersoy Yalçın, Sena Cihan Çağatay, Zeynep Büşra Aksoy, Duygu Ağagündüz, Bence Raposa

**Affiliations:** 1 Department of Nutrition and Dietetics, Institute of Health Sciences, Gazi University, 06560 Ankara, Türkiye; elif.akbas@marmara.edu.tr (E.A.); beyza.gurgen@afsu.edu.tr (B.N.G.); ece.akgul@kent.edu.tr (E.A.); esragunay@gazi.edu.tr (E.G.); burcaktok@gazi.edu.tr (B.T.); ecemooozduran@gmail.com (E.O.); dytsalihaersoy@gmail.com (S.E.Y.); senakir@outlook.com (S.C.Ç.); 2 Department of Nutrition and Dietetics, Faculty of Health Sciences, Marmara University, 34854 Istanbul, Türkiye; 3 Department of Nutrition and Dietetics, Faculty of Health Sciences, Afyonkarahisar Health Sciences University, 03030 Afyonkarahisar, Türkiye; 4 Department of Nutrition and Dietetics, Faculty of Health Sciences, İstanbul Kent University, 34406 Istanbul, Türkiye; 5 Stem Cell Institute, Ankara University, 06100 Ankara, Türkiye; zeynepbusraa@gmail.com; 6 Department of Nutrition and Dietetics, Faculty of Health Sciences, Gazi University, 06560 Ankara, Türkiye; 7 Institute of Basics of Health Sciences, Midwifery and Health Visiting, Faculty of Health Sciences, University of Pécs, 7622 Pécs, Hungary

**Keywords:** intestinal stem cells, diet, gut microbiota, nutrient sensing, epithelial regeneration

## Abstract

The intestinal epithelium is a dynamic tissue renewed by intestinal stem cells (ISCs) within the crypt niche. ISC behavior is regulated not only by intrinsic genetic programs but also by dietary composition, nutrient availability, microbial metabolites, and feeding rhythms. This narrative review synthesizes mechanisms through which diet influences ISC self-renewal, proliferation, differentiation, metabolic programming, and regenerative capacity. Dietary patterns exert context-dependent effects: caloric restriction, fasting, and structured feeding–fasting cycles may enhance epithelial regeneration through nutrient-sensing pathways, mitochondrial adaptation, and circadian regulation, whereas Western-type and high-fat diets may promote niche remodeling, inflammation, metabolic reprogramming, and tumorigenic risk. Macronutrients regulate ISC fate through carbohydrate metabolism, amino acid sensing, fatty acid oxidation, and lipid-derived signaling molecules. Micronutrients, including vitamins A, D, and B and minerals such as iron, zinc, selenium, and magnesium, contribute to epithelial differentiation, redox balance, and barrier integrity. Phytochemicals may modulate ISC function through Wnt/β-catenin, Nrf2, SIRT1, and epigenetic pathways or by stabilizing the epithelial microenvironment. The gut microbiota mediates diet–ISC interactions via short-chain fatty acids, bile acids, indole derivatives, and other metabolites. However, human evidence remains limited, and studies integrating human organoids, single-cell analyses, metabolomics, and chrononutrition are needed to inform personalized nutritional strategies for intestinal health.

## 1. Introduction

The intestinal epithelium constitutes the largest mucosal surface of the human body. It performs highly specialized functions, including nutrient digestion and absorption, maintenance of fluid–electrolyte balance, and the formation of an effective barrier against pathogens [1,2]. Due to continuous exposure to luminal contents and a high metabolic workload, this dynamic tissue undergoes complete renewal approximately every 3–5 days [2,3]. This remarkable regenerative capacity is sustained by intestinal stem cells (ISCs), located at the base of the crypts, which maintain epithelial integrity through continuous proliferation and differentiation [1,4].

The ISC hierarchy consists of two functionally distinct populations: actively cycling leucine-rich repeat-containing G protein-coupled receptor 5-positive cells (Lgr5^+^) crypt base columnar (CBC) cells, which are responsible for maintaining epithelial homeostasis under basal conditions, and more quiescent “reserve” stem cells, located at the +4 position and characterized by markers such as HOP homeobox^+^, B lymphoma Mo-MLV insertion region 1 homolog (Bmi1^+^), and mouse telomerase reverse transcriptase (mTert^+^), which are activated in response to stress conditions such as radiation, inflammation, or tissue injury [3,5,6]. This hierarchical model has been established primarily through lineage-tracing and genetic mouse studies [7]; the extent to which an analogous reserve population exists and behaves similarly in the human intestinal epithelium remains incompletely defined [8]. The functional balance between these two populations is a critical determinant of both epithelial maintenance and regenerative capacity [2].

ISC behavior is tightly regulated by a complex microenvironment known as the stem cell niche, which comprises Paneth cells, mesenchymal telocytes, stromal cells, and immune cells [4,9]. This niche provides key regulatory signals, including Wnt, Notch, bone morphogenetic protein (BMP), epidermal growth factor (EGF), and Hedgehog pathways, which collectively orchestrate the molecular mechanisms governing stem cell self-renewal and lineage differentiation into specialized epithelial cell types such as enterocytes, goblet cells, and enteroendocrine cells [9,10,11]. Disruption of this finely tuned signaling network has been closely associated with the pathogenesis of intestinal disorders, including inflammatory bowel disease and colorectal cancer [9,12,13]. These regulatory circuits have been delineated largely through murine genetic models and intestinal organoid systems [7,10,11]; corresponding evidence from human tissue remains comparatively sparse [14].

Accumulating evidence indicates that diet functions not merely as a source of energy but also as a potent biological regulator, modulating ISC behavior through multiple interconnected mechanisms [12,15]. Nutritional status directly influences ISC hierarchy, metabolic programming, and regenerative capacity through nutrient-sensing pathways, including mechanistic target of rapamycin complex 1 (mTORC1), AMP-activated protein kinase (AMPK), SIRT1, and peroxisome proliferator-activated receptors (PPARs), mechanisms characterized predominantly in rodent and organoid models [3,4,12]. Collectively, these findings position ISCs as highly dynamic entities shaped not only by intrinsic genetic programs but also by metabolic and environmental cues [2,4].

Converging evidence from experimental and translational studies demonstrates that distinct dietary patterns exert significant and context-dependent effects on ISC biology [1,2]. Energy-restrictive interventions, such as CR and short-term fasting, have been shown to preserve stem cell function and enhance tissue regeneration, partly by suppressing mTORC1 activity and expanding injury-resistant reserve ISC populations [5,12,16]. In contrast, Western-type diets characterized by high fat and sugar intake have been associated with disruption of the stem cell niche, induction of inflammatory responses, metabolic reprogramming, and an increased risk of colorectal tumorigenesis [4,12,17]. Collectively, these observations underscore the non-linear, dose-dependent, and context-specific nature of dietary influences on ISC function [1,17]. In addition to overall dietary patterns, both macronutrients and micronutrients, including vitamins, minerals, and trace elements, contribute to the regulation of ISC proliferation, differentiation, and survival [3,18]. Furthermore, bioactive dietary compounds, such as polyphenols and other phytochemicals, may influence long-term stem cell programming through epigenetic and metabolic mechanisms, highlighting an additional layer of dietary regulation [1,19].

A critical mediating interface in the diet–stem cell interaction is the gut microbiota [2,9]. Microbial fermentation of dietary components generates a wide range of bioactive metabolites, including short-chain fatty acids (SCFAs), indole derivatives, lactate, and secondary bile acids, which can modulate ISC behavior both directly and indirectly through immune-mediated mechanisms [6,11,18]. This complex interplay is increasingly conceptualized as the “diet–microbiota–ISC axis,” an important regulatory system governing intestinal homeostasis [9,11].

Notwithstanding these advances, current knowledge remains predominantly derived from animal and organoid models, with direct human evidence remaining limited in scope and methodologically heterogeneous [17,18,20,21]. Specifically, the molecular markers, niche signals, and nutrient-sensing pathways summarized above have been characterized almost exclusively in genetically engineered mice and murine-derived organoids; where human data exist, they typically originate from intestinal biopsy-derived organoids or observational cohorts and are constrained by small sample sizes, short follow-up periods, and substantial inter-individual variability [22]. Furthermore, the context-dependent and multi-layered nature of diet–microbiota–stem cell interactions considerably complicates mechanistic interpretation and cross-study comparability. Consequently, a comprehensive and integrative synthesis of these interactions is critically needed to bridge the existing translational gap [17,18].

**Figure 1:**
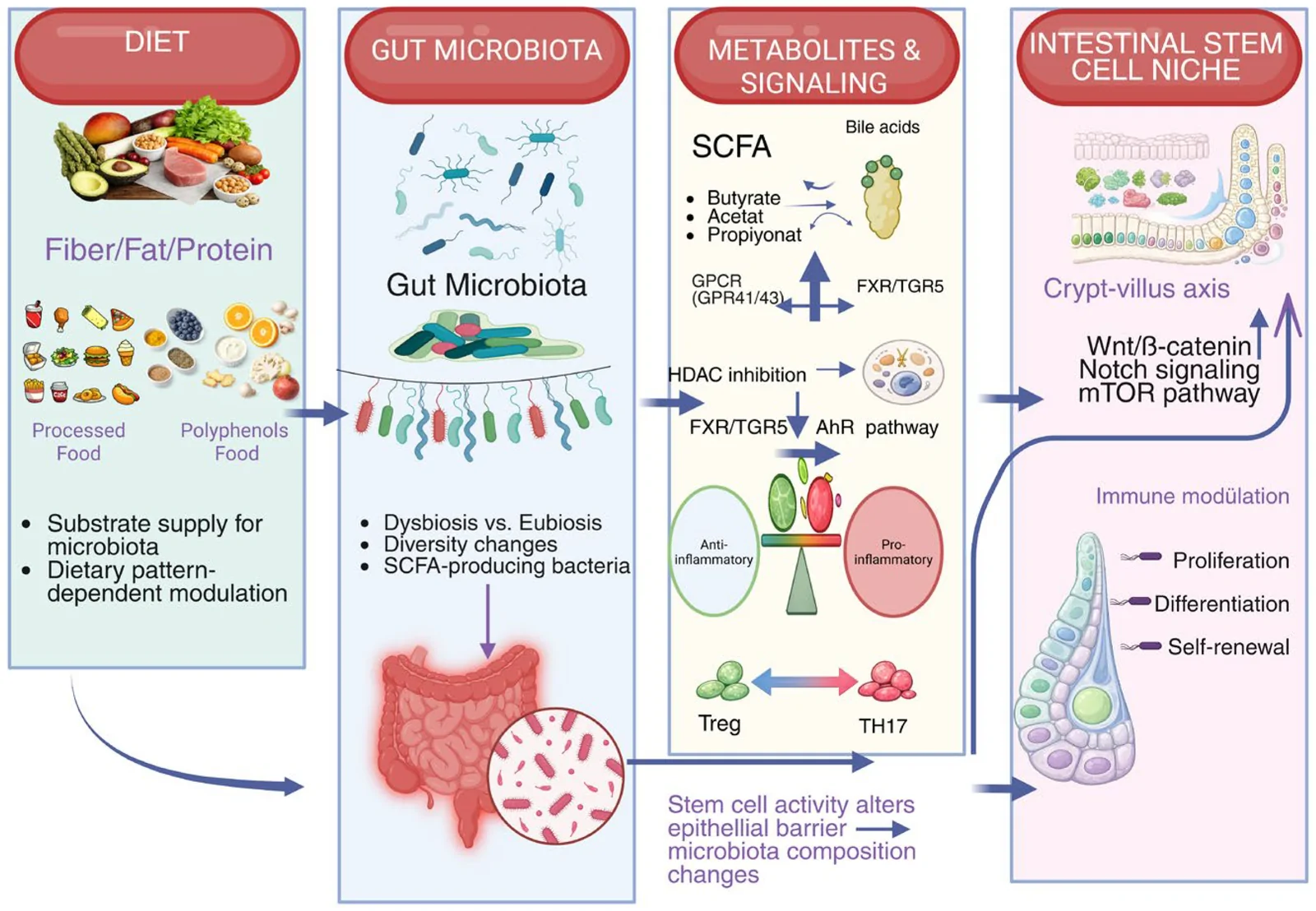
The diet–microbiota–intestinal stem cell interaction axis. Abbreviations: AhR, aryl hydrocarbon receptor; FXR, farnesoid X receptor; GPCR, G protein-coupled receptor; GPR41/43, G protein-coupled receptor 41/43; HDAC, histone deacetylase; mTOR, mechanistic target of rapamycin; SCFA, short-chain fatty acid; TGR5, Takeda G protein-coupled receptor 5; TH17, T helper 17 cells; Treg, regulatory T cells; Wnt/β-catenin, Wnt/β-catenin signaling pathway. Created in Biorender. Ersoy Yalçın, S. (2026) https://BioRender.com/s6b32fc, accessed on 13 August 2026.

Against this background, the present narrative review aims to provide a critical and integrative evaluation of how dietary patterns—encompassing CR, ketogenic diets (KDs), and high-fat diets—alongside macronutrients, micronutrients, and microbiota-derived metabolites, modulate ISC behavior. Elucidating these mechanisms may ultimately inform the development of evidence-based strategies to preserve intestinal homeostasis, attenuate aging-related epithelial decline, and advance stem cell–targeted personalized nutrition interventions [17]. The diet–microbiota–ISC interaction axis and its key regulatory components are summarized in Figure 1.

## 2. Intestinal Stem Cell Biology: Concepts Relevant to Nutrition

The intestinal epithelium forms a single layer of specialized cells that line the small and large intestines. It performs essential functions in nutrient absorption, fluid and electrolyte balance, immune regulation, and protection against microorganisms [23,24,25,26]. Continuous exposure to dietary components, microbial metabolites, and luminal antigens requires rapid epithelial turnover to maintain barrier integrity [27]. This renewal is sustained by ISCs, which reside at the base of intestinal crypts and maintain epithelial homeostasis, repair tissue damage, and contribute to intestinal tumorigenesis [7,27,28].

ISCs divide to generate transient amplifying (TA) progenitors that proliferate before differentiating into the major epithelial lineages of the small intestine, including absorptive enterocytes and secretory cells such as goblet, enteroendocrine, tuft, and Paneth cells [29,30,31]. Most epithelial cells migrate toward the villus tip, where they undergo apoptosis and are shed within three to five days. In contrast, Paneth cells migrate downward and remain at the crypt base for several weeks [27,32]. This continuous cycle of proliferation, differentiation, migration, and cell loss maintains epithelial integrity and intestinal function [27].

**Figure 2:**
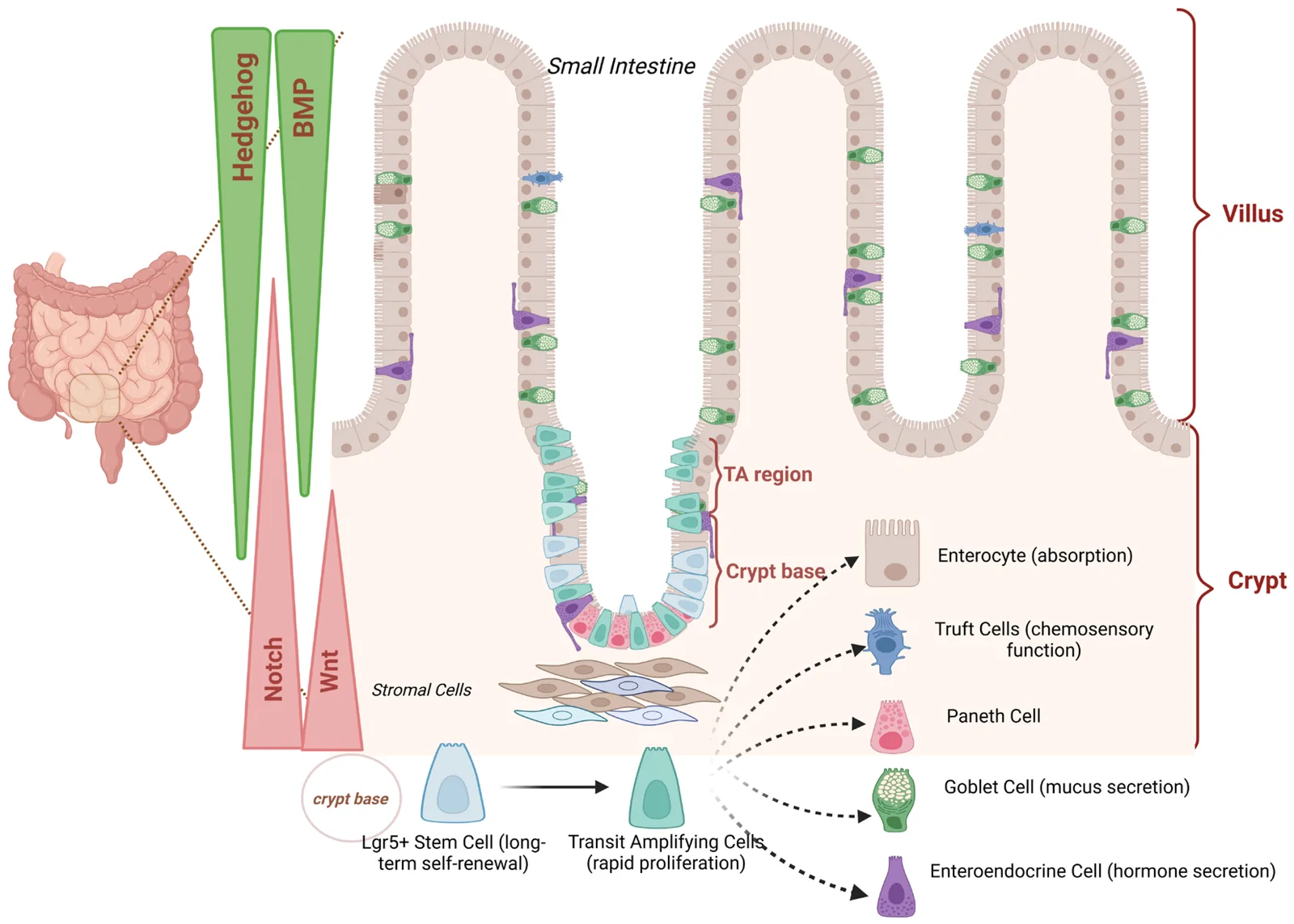
Structural organization of the intestinal stem cell niche. Abbreviations: BMP, bone morphogenetic protein; Lgr5^+^, leucine-rich repeat-containing G protein-coupled receptor 5-positive cells; TA, transient amplifying; Wnt, wingless-type signaling pathway. Created in BioRender. Günay, E. (2026) https://BioRender.com/1b7vnbk, accessed on 13 August 2026.

ISCs exhibit remarkable cellular plasticity, enabling dynamic adjustment of self-renewal and lineage commitment in response to physiological demands and environmental cues. These processes are regulated by multiple signaling pathways, including Wnt, Notch, Hippo, EGF, and BMP signaling [33,34]. The structural organization of the intestinal stem cell niche is illustrated in Figure 2.

### 2.1. Intestinal Stem Cell Niche and Homeostasis

Intestinal crypts contain distinct stem cell populations that cooperate to maintain epithelial renewal. The primary drivers of homeostatic epithelial turnover are actively cycling CBC stem cells characterized by high expression of Lgr5 [35]. A more quiescent stem cell population located around the +4 position above the crypt base expresses markers such as Bmi1 and mTert and functions as a reserve regenerative pool activated during mucosal injury [36,37,38,39,40].

Recently, fibroblast growth factor-binding protein 1 (Fgfbp1)–positive cells of the upper crypt have been identified as a source of epithelial regeneration, capable of reconstructing the crypt–villus axis, including the Lgr5^+^ CBC compartment, through biphasic regenerative trajectories [41,42]. Rather than reflecting a rigid division of labor between a fixed active pool and a dedicated quiescent reserve, such findings indicate that regenerative capacity is largely a plastic, facultative property. Upon injury, committed progenitors and even differentiated epithelial cells can re-acquire stem-cell function and replenish the Lgr5^+^ compartment [42,43,44]. This cellular plasticity, rather than strict redundancy between two predefined populations, underlies the regenerative resilience of the intestinal epithelium [18].

#### 2.1.1. Mesenchymal Niche

The mesenchymal compartment surrounding intestinal crypts contains diverse cell populations, including fibroblasts, myofibroblasts, endothelial cells, smooth muscle cells, and pericytes that provide both structural support and regulatory signals [14,45]. Among these, mesenchymal cells expressing the transcription factor forkhead box L1 (Foxl1) largely overlap with platelet-derived growth factor receptor α (PDGFRα)-positive telocytes and produce niche-supporting molecules such as Wnt2b and R-spondin 3 that sustain ISC maintenance [46,47].

Stromal populations, including PDGFRα^+^/cluster of differentiation 81^+^ trophocytes, further maintain a permissive stem cell environment by secreting BMP antagonists such as Noggin and Gremlin 1 (Grem1) [48,49]. These gradients establish a low-BMP environment at the crypt base that permits stem cell activity while promoting differentiation toward the villus region. Additional stromal components, including lymphatic endothelial cells and Grem1-expressing fibroblasts, produce R-spondin 3 and REELIN to support ISC maintenance during both homeostasis and tissue repair [34,50,51,52,53]. Collectively, these mesenchymal signals create a dynamic microenvironment that coordinates stem cell proliferation and differentiation.

#### 2.1.2. Epithelial Niche

ISCs are also regulated by specialized neighboring epithelial cells that form their immediate niche within the crypt. Paneth cells located adjacent to Lgr5^+^ stem cells secrete essential niche factors, including Wnt3a, EGF, transforming growth factor-α, and Notch ligands such as DLL4 [54,55,56]. These signals promote stem cell proliferation and maintain the undifferentiated state of CBC stem cells.

The epithelial niche demonstrates substantial plasticity during tissue repair. Following depletion of Lgr5^+^ ISCs, several differentiated epithelial cell types can dedifferentiate and reacquire stem-like properties, contributing to epithelial regeneration and restoration of crypt–villus architecture [42,43,44]. Secretory epithelial cells, including enteroendocrine and tuft cells, can also provide Notch signaling required for maintenance of the stem cell compartment [44].

Today, we know that the severe injury engages a transient, regeneration-specific program in which surviving epithelial cells adopt a fetal-like stem-cell state. A rare, normally quiescent clusterin-positive (Clu^+^) population, which is also termed revival stem cells (revSCs), is induced upon injury and transiently stimulates and regenerates Lgr5^+^ CBCs, as a result restoring the conventional hierarchy [57]. This repair-focused state is driven largely by YAP/TAZ-dependent reprogramming, which transiently dampens the canonical Wnt/Lgr5 program in favor of a fetal-like transcriptional signature that supports wound healing [58,59]. Fatty acid oxidation, mTORC1 activity, and redox state converge on precisely these injury-responsive, YAP-linked circuits. Consequently, the diet–regeneration interface may operate as much through facultative/revival programs as through homeostatic Lgr5^+^ stem cells.

### 2.2. Stem Cell Fate Decisions and Signaling Pathways

The fate of ISCs is governed by coordinated intracellular signaling pathways. Wnt signaling represents a central driver of ISC maintenance and proliferation within the crypt base [46]. Wnt ligands derived from both mesenchymal cells and Paneth cells bind to the Frizzled–LRP5/6 receptor complex, resulting in stabilization and nuclear translocation of β-catenin. β-catenin subsequently interacts with T-cell factor/lymphoid enhancer-binding factor (TCF/LEF) transcription factors to activate genes that maintain stem cell identity.

In contrast, the BMP signaling pathway restricts excessive stem cell proliferation and promotes differentiation along the crypt–villus axis [47]. BMP ligands produced by epithelial and stromal cells activate epithelial BMP receptors to drive terminal differentiation, while gradients of BMP antagonists such as Gremlin 1 and Noggin maintain a permissive stem cell environment at the crypt base [34,60].

Notch signaling cooperates with Wnt signaling to regulate lineage commitment. Activation of Notch through ligands Delta-like canonical Notch ligand 1 (DLL1) and DLL4 results in nuclear translocation of the Notch intracellular domain and induction of hairy and enhancer of split 1 (HES1), which suppresses the atonal bHLH transcription factor 1 (ATOH1) and directs progenitor cells toward the absorptive enterocyte lineage rather than secretory differentiation [61].

EGF signaling also contributes to ISC proliferation by activating downstream pathways that promote epithelial growth and regeneration. EGF secreted by Paneth and stromal cells is essential for intestinal organoid growth and ISC expansion [32,62].

**Figure 3:**
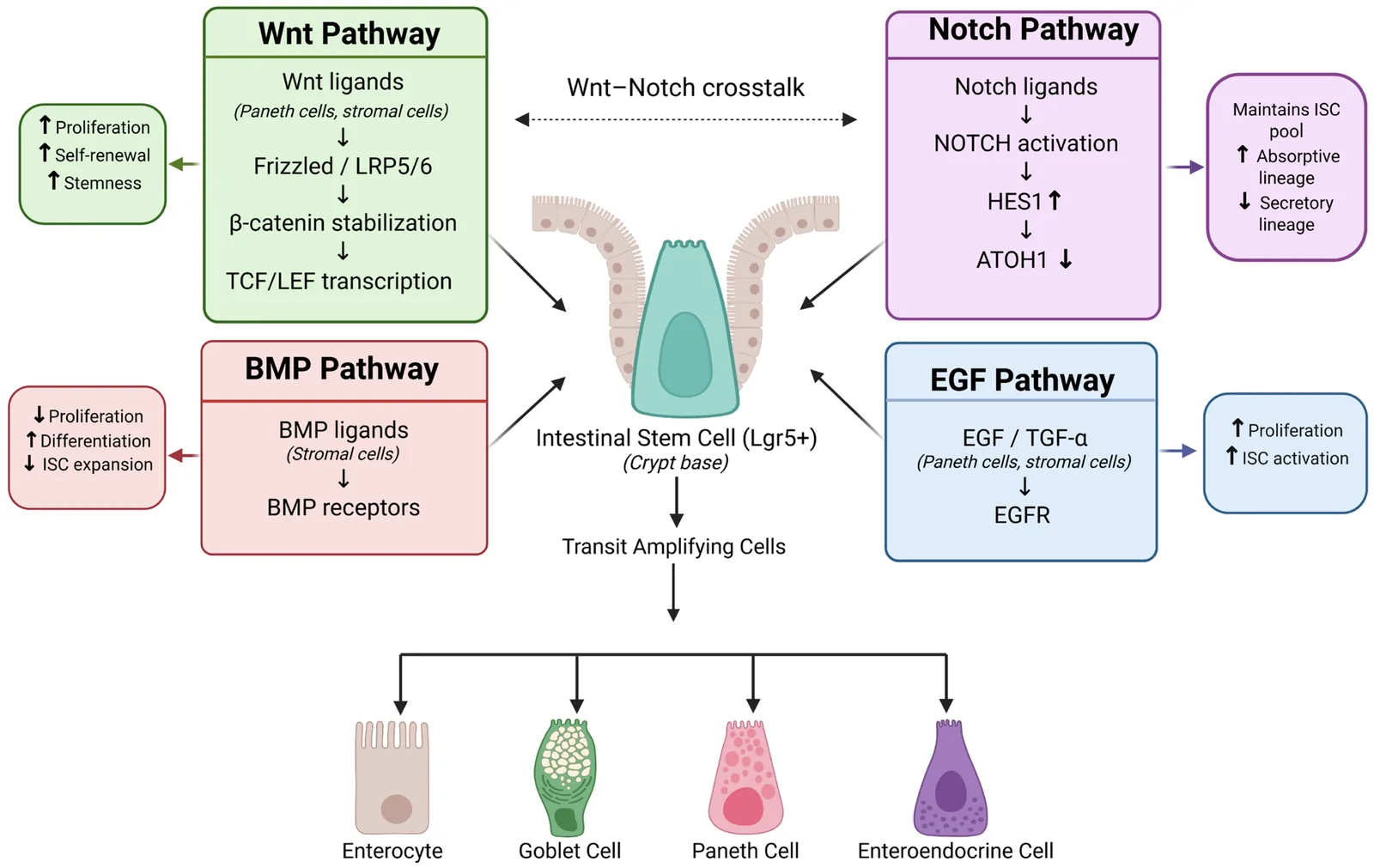
Key signaling pathways regulating ISC fate. Wnt, Notch, BMP, and EGF signaling pathways coordinately regulate ISC self-renewal, proliferation, and differentiation within the crypt niche. Wnt and EGF signaling promote ISC maintenance and proliferation, whereas BMP signaling inhibits ISC expansion and promotes differentiation. Notch signaling regulates lineage specification by favoring absorptive cell differentiation and suppressing secretory lineages. These pathways integrate signals from the niche to maintain intestinal epithelial homeostasis. Abbreviations: ATOH1, atonal bHLH transcription factor 1; BMP, bone morphogenetic protein; EGF, epidermal growth factor; EGFR, epidermal growth factor receptor; HES1, hairy and enhancer of split 1; ISC, intestinal stem cell; Lgr5^+^, leucine-rich repeat-containing G protein-coupled receptor 5-positive cells; LRP5/6, low-density lipoprotein receptor-related protein 5/6; TCF/LEF, T-cell factor/lymphoid enhancer-binding factor; TGF-α, transforming growth factor alpha; Wnt, wingless-type signaling pathway. Created in Biorender. Akgül, E. (2026) https://BioRender.com/wuiktcp, accessed on 13 August 2026.

The Hippo signaling pathway regulates epithelial architecture and regenerative capacity through its downstream effectors Yes-associated protein (YAP) and transcriptional coactivator with PDZ-binding motif (TAZ). Under homeostatic conditions, these proteins are inactivated by phosphorylation; however, nuclear YAP/TAZ interacts with TEA domain transcription factor (TEAD) transcription factors to promote expression of proliferation-associated genes such as MYC and connective tissue growth factor (CTGF) [52,59,63]. Activation of YAP expands the proliferative compartment along the crypt–villus axis and plays a critical role in epithelial regeneration following injury. These key signaling pathways governing ISC fate decisions are summarized in Figure 3.

### 2.3. Nutrient-Sensing Pathways Regulating Intestinal Stem Cells

Because the intestinal epithelium directly interfaces with dietary nutrients and microbial metabolites, ISC activity is tightly linked to metabolic signaling networks. Nutrient-sensing pathways translate fluctuations in nutrient availability into intracellular programs that regulate stem cell proliferation and differentiation. Among these regulators, mechanistic target of rapamycin (mTOR), AMPK, sirtuin signaling, and lipid-sensing nuclear receptors such as peroxisome proliferator-activated receptors (PPARs) act as central metabolic hubs linking dietary signals to ISC fate [64,65,66].

mTOR complex 1 integrates amino acid availability with cellular growth, protein synthesis, and mitochondrial biogenesis. Activation of mTORC1 supports ISC expansion by promoting anabolic metabolism and biosynthetic pathways required for epithelial renewal [67,68]. While transient mTORC1 activation facilitates epithelial regeneration, chronic hyperactivation, as seen with aging or metabolic stress, can lead to stem cell exhaustion and impaired regenerative capacity [69,70].

The liver kinase B1 (Lkb1)–AMPK axis functions as a critical energy sensor that coordinates ISC activity during nutrient scarcity. In energy-restricted states, such as fasting or CR, adenosine triphosphate (ATP) depletion activates AMPK, a pathway essential for maintaining the proliferative capacity and organoid-forming potential of ISCs by coupling metabolic status to cellular expansion [64,71,72]. These two sensors are not isolated; they engage in cross-talk to balance ISC fate [73]. For instance, during energy stress or nutrient scarcity, AMPK suppresses mTORC1 signaling through multiple mechanisms: not only by directly phosphorylating Raptor and indirectly activating tuberous sclerosis complex 2 (TSC2) to induce growth arrest, but also by targeting FNIP1 and GATOR2 to modulate lysosomal biogenesis and glucose sensing [74,75]. Conversely, in nutrient-replete states, mTORC1 can inhibit AMPK by phosphorylating specific subunits to prevent its lysosomal targeting and LKB1-mediated activation [76]. This bidirectional feedback loop ensures a rapid and precise shift between energy-consuming anabolic growth and catabolic stem cell maintenance, priming ISCs for subsequent regeneration [77].

Sirtuins regulate histone and protein acetylation, thereby influencing mitochondrial function, cellular metabolism, and stem cell maintenance [78]. Sirtuin-1, a nicotinamide adenine dinucleotide (NAD^+^)-dependent deacetylase, links systemic energy status to ISC function [64,79]. Increased SIRT1 activity during CR supports ISC expansion and modulates differentiation of epithelial cell populations [64]. Mechanistically, the activity of sirtuins is interconnected with other nutrient-sensing pathways, most notably AMPK and mTORC1, establishing a regulatory network during energy stress [76]. During CR, Paneth cells within the intestinal crypt release cyclic ADP ribose to initiate a signaling cascade in LGR5^+^ ISCs [80]. This cascade activates SIRT1 and creates a unique functional convergence with mTORC1 that challenges the classical paradigm of CR [81]. SIRT1 deacetylates S6 kinase 1 (S6K1), which elegantly primes the substrate for subsequent phosphorylation and activation by mTORC1 [81,82].

To sustain this regenerative activity, LGR5^+^ CBCs exhibit a distinct metabolic phenotype characterized by high mitochondrial oxidative phosphorylation, whereas the neighboring Paneth cells are more glycolytic and supply lactate as fuel [65,83]. This metabolic specialization supports the rapid proliferation and biosynthetic demands of the stem cell compartment [21,83]. Accordingly, the restoration of NAD^+^ levels through precursors such as nicotinamide riboside or nicotinamide mononucleotide has been shown to enhance mitochondrial homeostasis and improve the organoid-forming capacity of ISCs [84,85,86].

Lipid-sensitive transcription factors further integrate dietary signals with stem cell behavior. Peroxisome PPARs regulate fatty acid oxidation and transcriptional programs required for stem cell maintenance [87]. High-fat diets enhance ISC self-renewal by activating PPAR-dependent fatty acid oxidation. This metabolic shift generates intermediate metabolites, such as acetyl-CoA, that promote epigenetic changes to stabilize β-catenin and amplify Wnt target gene expression, thereby driving robust ISC proliferation [64,88]. Conversely, dietary sugars may influence lineage specification by modulating interactions between Wnt and Notch signaling pathways [88,89,90]. Furthermore, nutritional interventions can target this axis: Highland barley β-glucan stimulates PPAR-α-mediated ISC proliferation via docosahexaenoic acid, and natural flavonoid glycosides have been shown to rejuvenate organoid growth in aged models by modulating the PPAR-γ/epidermal growth factor receptor balance [17,91,92].

**Figure 4:**
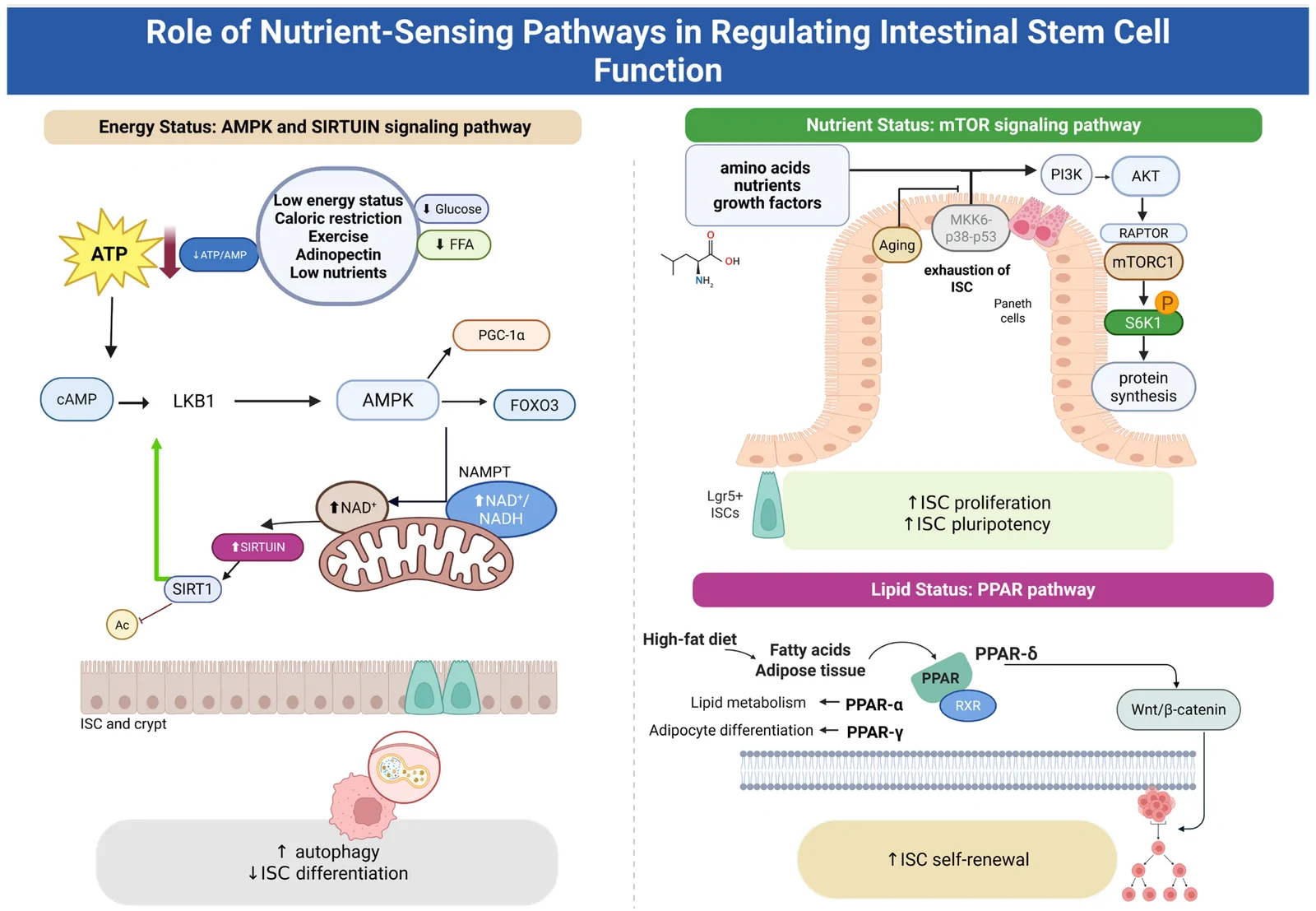
Role of nutrient-sensing pathways in regulating intestinal stem cell function. Abbreviations: AKT, AKT serine/threonine kinase/protein kinase B; AMPK, AMP-activated protein kinase; ATP, adenosine triphosphate; cAMP, cyclic adenosine monophosphate; FFA, free fatty acids; FOXO3, forkhead box O3; ISC, intestinal stem cell; ISCs, intestinal stem cells; Lgr5^+^, leucine-rich repeat-containing G protein-coupled receptor 5-positive cells; LKB1, liver kinase B1; mTOR, mechanistic target of rapamycin; mTORC1, mechanistic target of rapamycin complex 1; NAD^+^, nicotinamide adenine dinucleotide; NADH, reduced nicotinamide adenine dinucleotide; NAMPT, nicotinamide phosphoribosyltransferase; PGC-1α, peroxisome proliferator-activated receptor gamma coactivator 1-alpha; PI3K, phosphoinositide 3-kinase; PPAR, peroxisome proliferator-activated receptor; PPARα, peroxisome proliferator-activated receptor alpha; PPARγ, peroxisome proliferator-activated receptor gamma; PPARδ, peroxisome proliferator-activated receptor delta; RAPTOR, regulatory-associated protein of mTOR; RXR, retinoid X receptor; S6K1, ribosomal protein S6 kinase beta-1; SIRT1, sirtuin 1; ULK1, unc-51-like autophagy activating kinase 1; Wnt/β-catenin, Wnt/β-catenin signaling pathway. Created in BioRender. OZDURAN, E. (2026) https://BioRender.com/8sfhmbe, accessed on 13 August 2026.

In summary, the AMPK–mTOR, NAD^+^/SIRT1, and PPAR/FAO pathways form a highly integrated signaling matrix to execute epithelial regeneration. Specifically, energy stress activates AMPK, which simultaneously suppresses mTORC1 to enforce catabolic stem cell maintenance and elevates intracellular NAD^+^ levels to stimulate SIRT1, which consequently optimizes mitochondrial oxidative phosphorylation [76,86,93]. Concurrently, PPARδ efficiently channels dietary or stored lipid substrates into this network via fatty acid oxidation, while independently co-activating β-catenin to amplify Wnt signaling [88,94]. This synchronized metabolic and transcriptional shift is precisely what provides both the metabolic and self-renewal signals required to support epithelial regeneration [81]. The nutrient-sensing pathways linking dietary signals to ISC function are summarized in Figure 4.

## 3. Intestinal Stem Cells and Dietary Patterns

The current literature demonstrates that dietary patterns reshape ISC biology through multiple mechanisms [95]. While a substantial portion of existing studies has focused on the effects of Western-type and KD, interest in the regulatory and potentially protective effects of plant-based and Mediterranean-type dietary patterns on ISCs has been increasing in recent years [96,97,98]. Within this framework, it is understood that diet influences ISC functions primarily through two main mechanisms.

The first mechanism involves the metabolic programming of crypt cells and the regulation of epithelial energy balance [18]. The balance between lipid and carbohydrate metabolism, mitochondrial functions, cellular responses to hypoxia, and signaling pathways associated with ketone bodies plays a critical role in determining ISC behavior [88]. In particular, Western-type (high-fat, high-sugar) dietary patterns and KD are extensively investigated due to their strong metabolic effects and their influence on ISC proliferation, differentiation dynamics, and tumorigenesis [99,100]. In this context, macronutrient composition and ratios define the fundamental metabolic environment that shapes the proliferative capacity, differentiation tendency, and stress responses of stem cells [99,101].

The second mechanism is the multilayered interaction network between the ISC niche, the immune system, and the microbiota [102]. Immune signals regulated by pattern recognition receptors (PRRs) and cytokines such as interferon gamma (IFN-γ) and interleukin-22 (IL-22) [103,104], along with alterations in microbiota composition and microbiota-derived metabolites such as SCFAs and bile acids, play a decisive role in regulating ISC functions [105]. These interactions directly affect not only stem cell activity but also epithelial integrity, inflammatory responses, and tissue regenerative capacity [99,101].

A review of the current literature indicates that the strongest causal evidence for the relationship between dietary patterns and ISC functions derives from animal models and organoid-based experimental systems, which enable direct mechanistic investigation of ISC behavior under defined nutritional conditions [95,96]. In contrast, available human evidence is largely based on epidemiological, clinical, and molecular association data, while biopsy-derived or patient-derived functional models directly interrogating ISC activity remain comparatively limited [95]. This situation points to a significant knowledge gap in fully elucidating the implications of diet–ISC interactions in human physiology and in translating causal mechanisms identified in preclinical models to humans [95,106]. Therefore, advanced human studies integrating single-cell analyses, organoid-based functional models, and metabolomic approaches are needed to achieve a more comprehensive understanding of this relationship [107]. The main dietary interventions investigated in relation to ISC biology and their effects across experimental models are summarized in Table 1.

### 3.1. Western-Type Dietary Patterns

The Western-type diet is characterized by high saturated fat and refined sugar content, low fiber levels, and consumption of ultra-processed foods, and it can affect not only metabolic changes associated with energy excess in the intestinal epithelium but also the homeostasis of the ISC niche [97]. This dietary pattern can disrupt the balance of the microenvironment regulating ISC function by activating growth signaling pathways, particularly the insulin/IGF–AKT axis, while also altering microbiota-derived tonic signals and immune surveillance mechanisms. This condition may trigger an epithelial response characterized by proliferation, differentiation, and reprogramming of cellular programs within the crypts [101].

Experimental studies demonstrate that the effects of Western-type diets on ISC behavior are multidimensional [95]. In mice subjected to a high-fat–high-sugar diet, marked alterations in intestinal architecture have been observed, including increased villus length, changes in epithelial renewal rate, and enhanced proliferation in ISC and progenitor cell populations. Single-cell analyses have revealed accelerated cell cycling and shifts in differentiation trajectories during this process. In particular, increases in enterocyte and goblet cell populations have been observed, while reductions in certain enteroendocrine cell subtypes have been reported [96,101,113].

Not all structural adaptations to Western-type dietary components appear to be driven by increased ISC or progenitor proliferation. In the study by Taylor et al., which examined high-fructose consumption, mice fed a high-fructose corn syrup diet exhibited a 25–40% increase in duodenal and proximal jejunum villus length, accompanied by increased intestinal surface area and lipid absorption. This increase was reported to be associated with prolonged epithelial cell survival rather than increased proliferation. Thus, modulation of the crypt–villus axis and reduced epithelial cell loss may represent an additional adaptive mechanism through which Western-type dietary components enhance intestinal absorptive capacity, at least in murine models [96].

In addition to these structural adaptations, preclinical evidence indicates that Western-type diets may also influence ISC function through alterations in the intestinal immune microenvironment [95,99]. In murine models, high-fat diet feeding was shown to suppress major histocompatibility complex (MHC) class II expression in intestinal epithelial cells, including Lgr5^+^ ISCs, in association with reduced intestinal microbial diversity. Microbiome-manipulation experiments further supported a role for the intestinal microbiota in regulating epithelial MHC-II expression. Importantly, in Apc-deficient murine models, MHC-II− Lgr5^+^ ISCs exhibited greater tumor-initiating capacity than their MHC-II+ counterparts, suggesting that HFD-induced suppression of MHC-II may facilitate tumor initiation by weakening epithelial immune surveillance [99].

From an integrative perspective, the effects of Western-type diets on ISCs extend beyond enhanced proliferation and immune modulation and are closely linked to metabolic reprogramming of the stem cell compartment [95,108]. While several studies have reported increased ISC activity under high-fat or high-sugar dietary conditions [96,101,113], evidence from the New Western Diet (NWD1) model indicates that Western-type dietary exposures do not uniformly enhance canonical ISC activity and may instead alter stem cell identity and niche dynamics [108]. In NWD1-fed mice, mitochondrial function in Lgr5^+^ stem cells was suppressed, resulting in reduced canonical ISC activity. Simultaneously, alternative Bmi1^+^/Ascl2^hi stem-like populations were mobilized, indicating a compensatory adaptation within the epithelial renewal system. However, this adaptive response was accompanied by persistent low-grade inflammation and the establishment of a protumorigenic microenvironment, suggesting that Western dietary exposure may promote intestinal carcinogenesis not only through increased proliferation but also through stem cell reprogramming and niche remodeling [108].

Collectively, these apparently divergent ISC responses highlight the importance of the specific dietary and experimental context. Differences in fat and sugar sources, micronutrient and fiber content, duration of dietary exposure, and the resulting metabolic phenotype may contribute to the distinct ISC responses observed across Western-style dietary models [101,108]. Therefore, findings from individual experimental diets should not be generalized to Western dietary patterns as a whole, particularly given the limited direct evidence regarding ISC responses to these dietary patterns in humans [95,108].

### 3.2. High-Fat and Ketogenic Dietary Patterns

Preclinical evidence indicates that increased dietary fat intake can lead to significant structural and functional changes in the intestinal epithelium and ISC niche [95]. In an acute mouse model in which 60% of energy was derived from fat, HFD exposure rapidly increased intestinal epithelial proliferation and induced transcriptional changes in stem/progenitor populations, although crypt depth and villus height remained unchanged during the first week of exposure [97]. In longer-term HFD models, dietary fat has also been shown to alter ISC-associated immune surveillance, including suppression of MHC-II expression in Lgr5^+^ ISCs, which was linked to increased tumor-initiating capacity in an Apc-deficient context [99]. In addition, Western diet-induced Paneth cell dysfunction has been demonstrated in mice through microbiota-dependent DCA–FXR and type I IFN signaling; however, stem/progenitor proliferation was not directly altered in this model [109]. Given the central role of Paneth cells in the ISC niche, these findings suggest that high-fat and Western dietary exposures may influence ISC biology through both stem-cell-intrinsic and niche-mediated mechanisms, although the specific response appears to depend on dietary composition, exposure duration, and experimental model.

Experimental evidence further indicates that the transition to a high-fat diet triggers a rapid, dynamic adaptation in the intestinal epithelium. Within the first 24 h following dietary change, proliferative activity increases, and the expression of genes associated with cellular stress responses in stem/progenitor cells is elevated. Over the following days, the metabolic program shifts toward fatty acid metabolism, while transcriptional trajectory analyses suggest that progenitor cells become biased toward an absorptive enterocyte-like fate. Along with this process, lipid transport and absorption in enterocytes are also increased. These findings suggest that although the intestinal response to a high-fat diet initially appears adaptive, it may lead to metabolic and cellular reprogramming in the epithelium [97]. One mechanism underlying this reprogramming is fatty acid oxidation (FAO). Mechanistically, a high-fat diet activates the PPARα/δ-mediated FAO program in ISCs, thereby increasing stem cell number, proliferation, and function, which may support early tumorigenesis [88].

The effects of a high-fat diet are also shaped through interactions with the immune system and the microbiota [114]. It has been reported that this dietary model alters microbiota composition, weakens immune responses, and reduces the niche dependency of ISCs. This reduction in niche dependency may render stem cell function more autonomous and create a microenvironment that could contribute to the expansion of tumor-initiating cell populations [97].

The ketogenic diet (KD), although high in fat, is characterized by low carbohydrate intake and is therefore metabolically distinct from classical high-fat diets. This dietary model increases the production of ketone bodies, particularly β-hydroxybutyrate (BHB), thereby activating distinct signaling mechanisms in the intestinal epithelium [115]. It has been shown that ISCs can produce high levels of ketone bodies, and that these metabolites may regulate stem cell function through the Notch signaling pathway [116].

In addition, the effects of the KD on tumor biology differ markedly from those of classical high-fat diets. In an experimental study conducted by Dmitrieva-Posocco et al. [100], KD providing approximately 90% of energy from fat was shown to suppress colorectal tumor development. This effect was largely mediated by β-hydroxybutyrate, which signals through the hydroxycarboxylic acid receptor 2 (Hcar2) to increase the expression of the tumor suppressor gene Hopx. Through this mechanism, BHB suppresses Lgr5^+^ ISC and intestinal epithelial proliferation. The absence of a similar effect by acetoacetate suggests functional differences among ketone bodies [100]. These apparently contrasting effects of high-fat and ketogenic diets suggest that dietary fat content alone does not determine the ISC response. Differences in macronutrient composition, particularly carbohydrate availability and the consequent induction of ketosis, as well as fat composition, duration of dietary exposure, experimental model, metabolic status, and microbiota composition, may contribute to the divergent outcomes reported across studies [95,100]. Therefore, the effects observed with conventional high-fat diets should not be directly extrapolated to ketogenic diets, despite their similarly high fat content. Further studies, particularly in humans, are needed to determine the long-term implications of these distinct metabolic states for ISC function and tumorigenic risk.

### 3.3. Plant-Based and High-Fiber Dietary Patterns

Current evidence suggests that plant-based and high-fiber dietary patterns may influence ISC regulation primarily through microbiota-mediated metabolic processes and immune niche signaling. Short-chain fatty acids, produced by the gut microbiota during fermentation of dietary fibers, serve as both energy sources for epithelial cells and signaling molecules [102]. In addition, cytokines generated by interactions between the microbiota and mucosal immune cells are key regulators of ISC behavior, particularly within the crypt niche [110,111].

One of the prominent mechanistic studies in this field is the preclinical study by Corrêa et al. [110], which investigated dietary supplementation with the fermentable fiber inulin. In this experimental model, inulin consumption increased ISC proliferation in the colonic epithelium, leading to epithelial remodeling characterized by deeper crypt structures and an elongated colon. This effect was shown to be microbiota-dependent and was abolished under germ-free conditions. Furthermore, the absence of a similar phenotype in the absence of γδ T cells and IL-22 signaling indicates that high-fiber diets also regulate ISCs through the immune niche. The role of SCFAs in ISC regulation appears to be more complex. Although levels of acetate, propionate, and butyrate were increased with inulin consumption, the persistence of the epithelial response in the absence of the SCFA receptor free fatty acid receptor 2 (FFAR2) suggests that this effect cannot be explained through a single receptor. This finding indicates that the effects of high-fiber diets on ISCs are determined by a multi-component network involving microbiota composition, immune cell activation, and cytokine signaling [110].

Recent evidence indicates that the effects of high-fiber diets may vary depending on the biological context. The hypoxia-inducible factor 1 (HIF-1) signaling pathway has been reported to attenuate the increase in proliferation and stem cell-like properties associated with high-fiber diets. This finding indicates that high-fiber dietary patterns do not exert a unidirectional proliferative effect; rather, regulatory feedback mechanisms that preserve epithelial homeostasis are also engaged [111].

Overall, plant-based and high-fiber dietary patterns appear to create a microenvironment that supports intestinal epithelial renewal and barrier integrity. However, the long-term consequences of increased proliferative activity, particularly in relation to tumor development, remain to be fully elucidated. Moreover, direct evidence linking plant-based or high-fiber dietary interventions to ISC function in humans remains limited, as current mechanistic understanding is largely derived from preclinical models [110,111].

### 3.4. Mediterranean-Type Dietary Patterns

The Mediterranean dietary pattern is characterized by high consumption of monounsaturated fatty acids derived from olive oil, abundant intake of plant-based foods and dietary fiber, regular consumption of fish and omega-3 fatty acids, and low intake of refined sugars and processed meats [117]. Although studies directly examining the effects of the Mediterranean diet on ISCs are limited, existing evidence suggests that this dietary pattern may contribute to maintaining a more homeostatic intestinal microenvironment [98,112]. In particular, the balanced levels of microbiota-derived metabolites such as SCFAs and bile acids may contribute to the stability of the biochemical environment to which the crypt niche is exposed.

Preclinical evidence from a mucin-2-deficient murine model indicates that a Mediterranean-like fat blend can modulate intestinal inflammatory processes. Under isocaloric conditions, this dietary intervention reduced inflammation and disease severity. Given that inflammation is a significant stressor for the ISC niche in this model, where the mucus barrier is impaired, reducing the inflammatory burden may alleviate microenvironmental pressure on stem cells. However, the lack of direct assessment of ISC function in this study renders these interpretations indirect [112].

**Figure 5:**
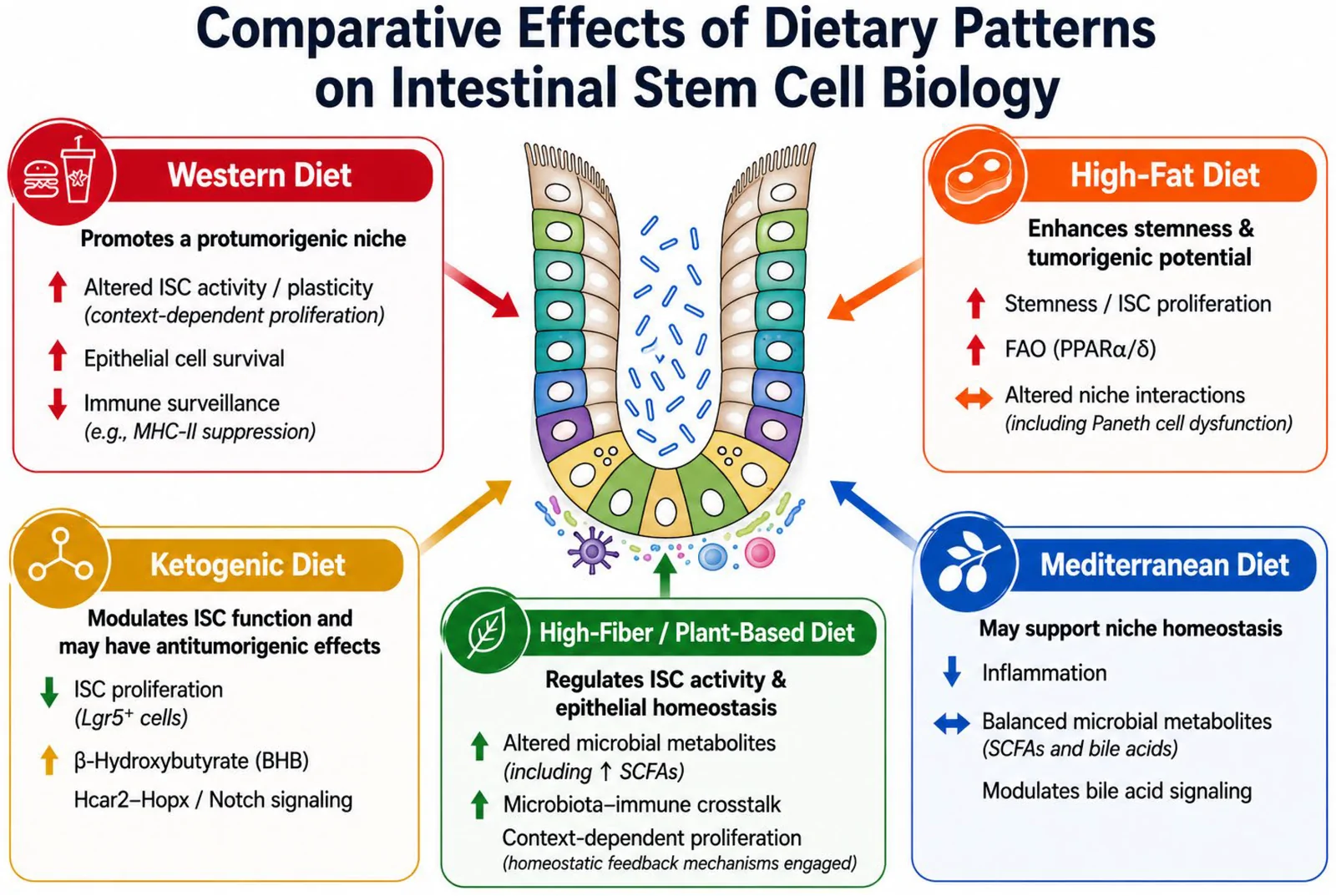
Comparative effects of dietary patterns on intestinal stem cell biology. The figure summarizes the major effects of Western-type, high-fat, ketogenic, high-fiber/plant-based, and Mediterranean dietary patterns on intestinal stem cell (ISC) activity and the surrounding niche. Western-type and high-fat diets are associated with context-dependent alterations in ISC proliferation and plasticity, epithelial cell survival, immune surveillance, fatty acid oxidation (FAO), and niche interactions, with some experimental models indicating increased tumorigenic potential. Ketogenic diets may exert distinct effects through β-hydroxybutyrate (BHB)-dependent signaling, including Hcar2–Hopx and Notch-related pathways, whereas high-fiber/plant-based diets influence ISC activity through microbiota-derived metabolites, microbiota–immune crosstalk, and context-dependent homeostatic responses. Mediterranean dietary patterns may support a more homeostatic intestinal environment through reduced inflammation and modulation of microbiota-derived metabolites and bile acid metabolism. Importantly, most ISC-specific mechanisms illustrated here are derived from preclinical mouse and/or organoid studies, whereas direct evidence in humans remains limited. Human studies, particularly those examining Mediterranean dietary interventions, primarily support changes in microbiota and metabolite profiles rather than direct effects on ISC function. Therefore, the illustrated pathways represent model-dependent mechanistic evidence and should not be interpreted as established causal effects in humans. The large color-coded arrows indicate the association of each dietary pattern with the intestinal crypt/ISC niche and correspond to the respective diet categories shown in the figure. Within each dietary-pattern panel, upward and downward arrows indicate reported increases and decreases, respectively, whereas bidirectional arrows indicate context-dependent, altered, or modulatory effects rather than a consistent unidirectional change. ISC, intestinal stem cell; FAO, fatty acid oxidation; PPAR, peroxisome proliferator-activated receptor; BHB, β-hydroxybutyrate; SCFAs, short-chain fatty acids.

Another important aspect of the Mediterranean diet that may be associated with ISC biology is bile acid metabolism. Findings from randomized controlled trials indicate that the effects of Mediterranean diet interventions may interact with the gut microbiota’s capacity to metabolize bile acids. Given that bile acids regulate metabolic and inflammatory signaling pathways in the intestinal epithelium via nuclear and membrane receptors, these metabolites are considered important mediators that may influence ISC behavior. However, interindividual differences in microbiota composition may modulate this response and contribute to heterogeneity in dietary effects [98]. The comparative effects of major dietary patterns on ISC biology are summarized in Figure 5.

## 4. Chrononutrition and Temporal Regulation of Intestinal Stem Cell Behavior

Circadian rhythms are endogenous approximately 24 h oscillations that coordinate physiological processes across multiple tissues, including the gastrointestinal tract. In the intestine, these rhythms regulate epithelial proliferation, metabolism, and tissue repair, thereby maintaining intestinal homeostasis [118]. The intestinal epithelium is one of the most rapidly renewing tissues in the body, and this process is driven by ISCs located at the base of intestinal crypts [119].

Accumulating evidence indicates that ISC behavior is closely linked to circadian clock mechanisms. These rhythms are generated by transcription–translation feedback loops involving core clock genes such as circadian locomotor output cycles kaput (CLOCK), brain and muscle ARNT-like 1 (BMAL1), PER, and cryptochrome circadian regulator (CRY), which regulate rhythmic gene expression in peripheral tissues [118]. Circadian clock components also interact with signaling networks involved in epithelial proliferation and differentiation, including Wnt, Notch, and Hippo pathways, providing a mechanistic link between temporal cues and stem-cell activity within the intestinal crypt niche [120].

Preclinical studies indicate that disruption of circadian regulation can alter intestinal epithelial homeostasis, although the direction and magnitude of these effects appear to depend on the experimental context. In an inducible mouse model with intestinal epithelial-specific Bmal1 deletion, loss of BMAL1 altered the response to chemically induced colonic inflammation rather than simply producing generalized impairment of intestinal homeostasis [121]. In contrast, a rat model exposed to constant light for four weeks demonstrated direct disruption of ISCs associated with sympathoexcitation, oxidative stress, and inhibition of Wnt5a signaling [122]. These findings underscore that the effects of circadian disruption on the intestinal epithelium may differ according to the clock component manipulated, the nature of the circadian disturbance, and the inflammatory or physiological context. Importantly, evidence directly linking environmental circadian disruption to human ISC dysfunction remains limited, and observations from experimental models should not be extrapolated directly to humans.

This circadian framework provides a biological basis for examining chrononutrition, particularly the effects of meal timing and feeding–fasting cycles on intestinal regeneration. The temporal distribution of food intake acts as a powerful synchronizer of peripheral circadian clocks, influencing metabolic pathways, immune responses, and gastrointestinal function. Evidence from chrononutrition research indicates that the timing of nutrient intake interacts closely with circadian rhythms to regulate gut physiology and microbial dynamics [123]. Disruptions in circadian alignment, such as irregular eating patterns or late-night meals, can alter microbial diversity, short-chain fatty acid production, and intestinal barrier integrity, thereby contributing to metabolic dysfunction [124]. However, these broader effects on gut physiology should be distinguished from direct effects on ISCs, which have been investigated predominantly in experimental models.

Feeding–fasting cycles may influence ISC behavior through the integration of nutrient availability, circadian signals, microbial metabolites, and cellular energy-sensing pathways. The intestinal epithelium undergoes continuous regeneration driven by ISCs, whose function is tightly regulated by nutrient-sensing pathways and metabolic signals [18]. Nutrient availability modulates ISC proliferation and differentiation through pathways such as insulin signaling and cellular metabolic regulation, highlighting the importance of dietary rhythms in maintaining epithelial integrity [125]. In aged mice, alternate-day fasting restored ISC number and regenerative capacity and improved intestinal epithelial function, with complementary organoid experiments implicating improved mitochondrial metabolism as an important mechanism [126]. These findings provide mechanistic evidence that feeding–fasting interventions can partly counter age-associated ISC dysfunction; however, whether comparable effects occur in the aging human intestine remains unknown.

Time-restricted feeding (TRF) and intermittent fasting (IF) encompass heterogeneous dietary strategies that differ in fasting duration, feeding-window length, caloric intake, and circadian alignment. Consequently, their biological effects should not be assumed to be equivalent. Meal timing serves as an important regulator of peripheral clock alignment and metabolic homeostasis [127,128,129], while changes in feeding schedules can modify gastrointestinal hormone secretion, microbial metabolism, and intestinal epithelial function. For example, a porcine TRF study demonstrated that restricted feeding altered gut microbial tryptophan metabolism and glucagon-like peptide-1 secretion [130]. Although such findings provide evidence that meal timing can modify the gut microbial–endocrine environment, this study did not constitute direct evidence of altered human ISC function.

More direct evidence for feeding-dependent regulation of ISC activity comes from experimental fasting–refeeding models. In mice, short-term post-fast refeeding produced a marked increase in Lgr5^+^ ISC-driven regeneration, with ex vivo organoid experiments supporting enhanced stem-cell function. Mechanistically, this response was mediated in part by robust mTORC1 activation and increased polyamine-dependent protein synthesis. Importantly, however, the same study demonstrated that the regenerative response was accompanied by increased tumorigenic potential: in the context of Apc loss, post-fast refeeding increased intestinal tumor formation [130]. Thus, enhanced ISC proliferation or regenerative capacity should not be interpreted as uniformly beneficial. The consequences of dietary stimulation of ISC activity are likely to depend on genetic background, tissue context, duration of exposure, and the presence of oncogenic alterations, emphasizing the need to balance regenerative benefits against potential long-term tumorigenic risk.

Evidence from other organisms further illustrates the model dependence of chrononutrition effects. In Drosophila melanogaster, TRF was associated with improved gut health and longevity without apparent fitness trade-offs [131]. However, findings from Drosophila cannot be assumed to reflect mammalian ISC biology directly and are better regarded as evidence that temporal feeding patterns can influence intestinal homeostasis across species.

Similarly, aging studies suggest that the response to fasting may vary according to species and intervention design. In a naturally aging mouse model, alternate-day fasting improved mitochondrial metabolism and regenerative properties of ISCs [126], whereas a study in killifish found that aging reduced ISC activity and that IF partly reversed age-associated intestinal gene-expression patterns [132]. Together, these findings support a potential interaction between dietary timing and intestinal aging, but differences in species, fasting protocols, and outcome measures limit direct comparison across studies.

Feeding schedules may also influence epithelial lineage specification indirectly. In the porcine TRF model, changes in microbial metabolism and enteroendocrine signaling were observed [133], while enteroendocrine cells themselves have been shown to contribute to maintenance of the intestinal stem-cell niche through nutrient-responsive regulation of crypt metabolism [134]. These observations suggest a potential link between meal timing, microbial–endocrine signaling, and ISC niche regulation; however, direct causal evidence connecting TRF-induced enteroendocrine changes to ISC differentiation remains limited. Accordingly, established pathways such as Notch and Wnt should be viewed here as components of the broader ISC regulatory network described earlier rather than as independently demonstrated mediators of every chrononutrition intervention.

Studies outside the intestinal stem-cell compartment provide additional, but indirect, evidence that feeding rhythms can influence stem and progenitor cell biology. In an obese mouse model, TRF altered hematopoietic stem and progenitor cell activity in the bone marrow and reduced monocyte production [135]. In humans, a small prospective observational study of ten healthy men practicing Ramadan fasting reported an increase in circulating hematopoietic stem/progenitor cells [136]. However, hematopoietic and circulating progenitor cells are biologically distinct from ISCs; therefore, these findings should not be interpreted as evidence that fasting increases human intestinal stem-cell activity. Rather, they suggest that systemic metabolic responses to feeding–fasting cycles may extend to several stem-cell compartments and warrant tissue-specific investigation.

Despite the growing mechanistic evidence, several limitations complicate interpretation of the relationship between chrononutrition and ISC biology. Most studies directly examining ISC responses to circadian disruption, fasting, or refeeding have been conducted in rodents, organoids, Drosophila, killifish, or other experimental systems, whereas direct evidence from human intestinal tissue remains scarce. In addition, substantial heterogeneity exists in fasting duration, feeding-window length, intervention period, age, metabolic status, nutrient composition, and circadian alignment across studies. It is therefore often difficult to distinguish effects attributable specifically to meal timing from those resulting from caloric restriction, altered nutrient intake, weight change, metabolic adaptation, or microbiome remodeling. These methodological differences may also explain why apparently similar feeding interventions produce divergent regenerative outcomes across experimental settings.

**Figure 6:**
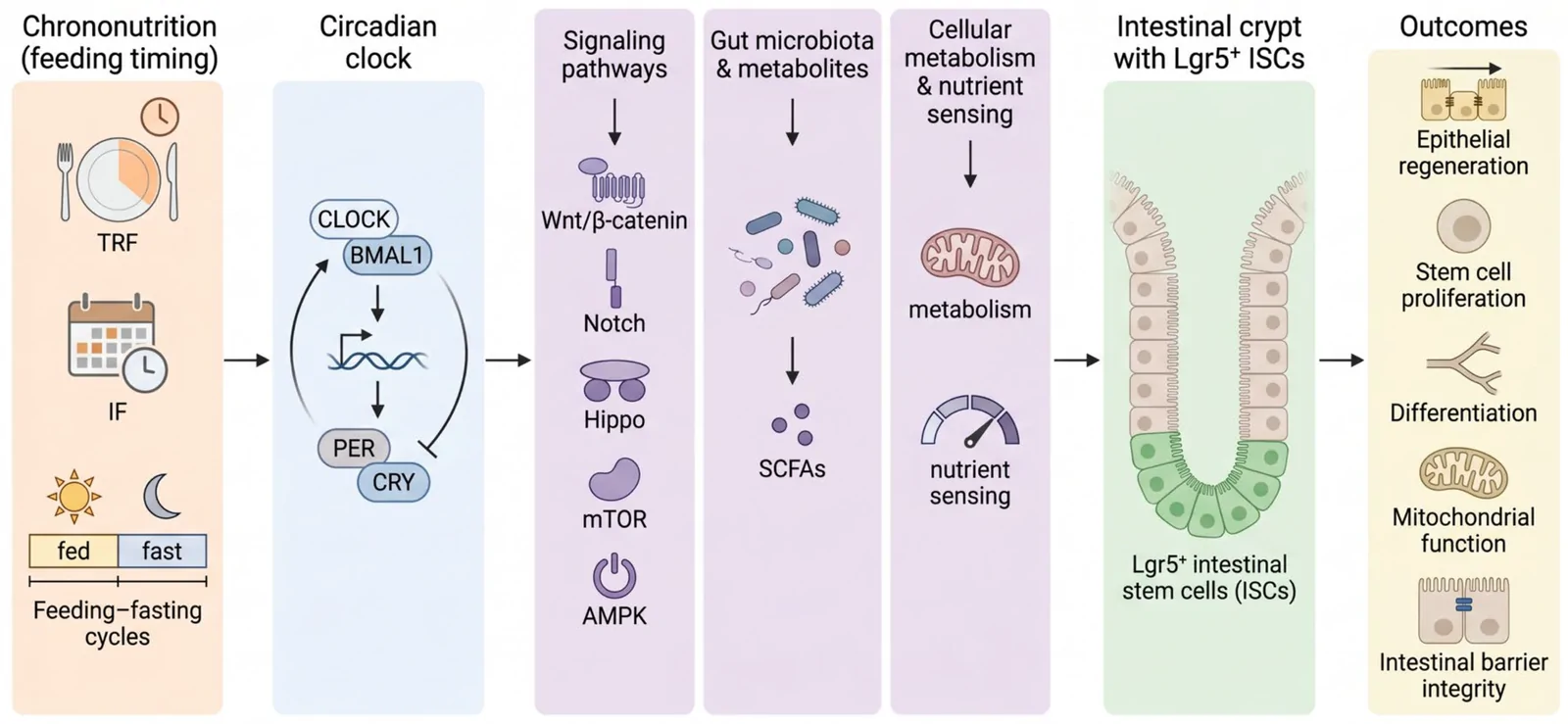
Chrononutrition and circadian regulation of intestinal stem cell activity. The mechanisms illustrated are based predominantly on preclinical animal and organoid studies. Abbreviations: AMPK, AMP-activated protein kinase; BMAL1, brain and muscle ARNT-like 1; CLOCK, circadian locomotor output cycles kaput; CRY, cryptochrome; IF, intermittent fasting; ISCs, intestinal stem cells; Lgr5^+^, leucine-rich repeat-containing G protein-coupled receptor 5-positive cells; mTOR, mechanistic target of rapamycin; PER, period; SCFAs, short-chain fatty acids; TRF, time-restricted feeding; Wnt/β-catenin, Wnt/β-catenin signaling pathway. Created in BioRender. Akbaş, E. (2026) https://BioRender.com/1ylk0ie, accessed on 13 August 2026.

Taken together, predominantly preclinical evidence indicates that feeding timing and feeding–fasting cycles can modulate intestinal physiology through coordinated effects on circadian regulation, microbial metabolism, nutrient sensing, mitochondrial function, and ISC activity. However, the magnitude, durability, and clinical relevance of these effects in humans remain uncertain. Future studies should distinguish the effects of meal timing from those of caloric restriction and nutrient composition and determine whether chrononutrition strategies can enhance epithelial repair while avoiding sustained or maladaptive stem-cell activation. The interactions among circadian timing, feeding–fasting cycles, and ISC activity are summarized in Figure 6.

## 5. Energy Intake, Macronutrients and Stem Cell Fate Decisions

ISC metabolism is highly plastic and responds dynamically to dietary and environmental cues. Within the crypt base, LGR5^+^ ISCs and neighboring Paneth cells display distinct but complementary metabolic profiles, characterized by higher mitochondrial activity in LGR5^+^ ISCs and greater reliance on glycolysis in Paneth cells. The LGR5^+^ stem cell pool exhibits high proliferative activity, which requires substantial energy [18,126]. Although LGR5^+^ cells exhibit high mitochondrial content, they have been reported not to depend predominantly on glucose oxidation [95,137]. This apparent dissociation between mitochondrial activity and glucose oxidation is consistent with the distribution of the mitochondrial pyruvate carrier (MPC), whose expression increases from the crypt base toward the villus. The crypt–villus gradient in MPC expression suggests position-dependent differences in mitochondrial pyruvate utilization [18,21,95]. Furthermore, recent data suggest that tricarboxylic acid (TCA) cycle metabolites and mitochondrial flux can influence the direction of ISC differentiation and its regenerative capacity [138]. Nevertheless, these mechanistic observations are derived predominantly from experimental models, and their direct relevance to human ISC metabolism remains insufficiently established.

Building on the nutrient-sensing mechanisms summarized in Section 2.3, energy-restriction models indicate that ISC responses are influenced by epithelial autophagy and by the distinct metabolic states associated with fasting and subsequent refeeding [95,139,140]. Using mouse and organoid models, Williams et al. (2023) provided evidence that the regenerative benefit of CR following irradiation depends on ATG7-dependent intestinal epithelial autophagy [140]. In a separate murine model of doxorubicin-induced intestinal injury, a 24 h fast was associated with reduced intestinal DNA damage, although the magnitude of this protection depended on the timing of post-fast refeeding. Fasting also increased the expression of genes associated with epithelial stem cells and DNA repair, suggesting an adaptive response to genotoxic injury [139]. By contrast, the post-fast refeeding phase elicits a biologically distinct response from fasting itself. In mice subjected to a 24 h fast followed by refeeding, post-fast refeeding enhanced ISC proliferation and regenerative capacity through mTORC1-dependent polyamine metabolism. In separate conditional Apc-deletion mouse models, however, it also increased intestinal tumor initiation. These findings indicate that enhanced ISC proliferation is not inherently beneficial, as the anabolic program supporting epithelial regeneration may also promote early neoplastic events in a genetically tumor-prone setting. Thus, the biological consequences of fasting–refeeding interventions may depend not only on the period of energy restriction but also on the timing and metabolic characteristics of subsequent refeeding, as well as the genetic context of the intestinal epithelium [130].

The effects of energy restriction are further modified by age and baseline metabolic status [126,141]. It has been reported that alternate-day fasting may support epithelial function and ISC-associated regenerative capacity by improving mitochondrial metabolism in the aged gut; furthermore, short-term dietary restriction initiated in later life may enhance the function of LGR5^+^ ISCs derived from aged mice and may be associated with certain epigenetic changes. These findings suggest that energy-restriction-based interventions also influence regenerative potential in the aging gut [126,141]. Collectively, these findings indicate that dietary restriction protocols cannot be regarded as biologically equivalent. Their effects appear to depend on the type, duration, and timing of restriction; the presence of epithelial injury; host age and metabolic status; and the characteristics of the subsequent refeeding period [126,141,142,143]. Because the available evidence is largely preclinical, these findings should not yet be interpreted as support for specific fasting or energy-restriction regimens in humans.

### 5.1. Dietary Carbohydrates

Enteroids are three-dimensional epithelial culture models derived from intestinal stem cells that enable the investigation of nutrient-responsive epithelial processes under controlled conditions. However, they only partially recapitulate the intestinal niche, as stromal, immune, microbial, and systemic influences are not fully represented [12]. The current literature indicates that the effect of carbohydrates on ISC biology is not uniform; the nature of the effect may vary depending on the type of carbohydrate, metabolic state, and fermentation products [1,6,17,21]. Highly proliferative cells in the crypt require a dynamic energy metabolism to produce ATP and synthesize new cellular biomass. In this context, glucose is regarded both as an energy-providing substrate and as a metabolic signal that can influence epithelial cell metabolism and proliferative programs [17]. However, increased carbohydrate availability or glycolytic activity does not necessarily indicate a beneficial regenerative response. Using murine and human colonoids and a high-sucrose–DSS mouse model, Burr et al. showed that short-term excess sucrose impaired post-injury proliferation of ISCs and transit-amplifying cells. Increased glycolysis without a parallel rise in aerobic respiration suggested impaired coupling to mitochondrial pyruvate oxidation [144]. Using genetic mouse models, organoids, and colorectal cancer cells, Li et al. showed that mTORC2–AKT–PFKFB2 signaling promotes glycolysis, ISC proliferation and self-renewal, and intestinal tumorigenicity [145]. These contrasting outcomes may reflect differences in exposure, injury status, metabolic context, and oncogenic background. Accordingly, glycolytic activation may support regeneration in some settings but promote pathological stem-cell expansion in others.

Beyond their direct metabolic effects, fermentable carbohydrates may influence ISC biology and epithelial differentiation through microbiota-derived metabolites. Microbial fermentation products of carbohydrates may influence epithelial differentiation in addition to ISC metabolism. In primary human enteroids derived from individuals with obesity, SCFA exposure promoted absorptive-lineage differentiation through HDAC inhibition, Notch/HES1 activation, and ATOH1 suppression, while reducing enteroid permeability [146]. However, these ex vivo findings should not be interpreted as direct evidence of an in vivo dietary effect, and their generalizability may be limited by the model and donor characteristics. As discussed in Section 3.3, evidence from experimental fiber-intervention models suggests that fermentable carbohydrates may influence ISC responses indirectly through microbiota–immune signaling. However, these effects should not be attributed exclusively to SCFAs or a single receptor pathway, as they appear to vary according to fiber type, microbial composition, and immune context [110].

### 5.2. Protein and Amino Acids

The rapidly renewing intestinal epithelium requires an adequate supply of essential amino acids to support high rates of protein synthesis. Available evidence has focused primarily on individual amino acids and amino acid deprivation, whereas the direct effects of total dietary protein intake on ISC function remain insufficiently characterized. Amino acids may also support proliferative metabolism by providing substrates for oxidative energy production and biosynthetic intermediates, including α-ketoglutarate [18,21,147]. Their effects on ISC maintenance and differentiation may nevertheless vary by amino acid. Using mouse enteroids and an in vivo murine model, Kozai et al. showed that valine deprivation reduced Lgr5^+^ ISC abundance, proliferation, and enteroid-forming capacity while increasing apoptosis. In contrast, isoleucine deprivation increased enteroendocrine cell abundance, suggesting distinct roles for individual branched-chain amino acids in ISC maintenance and differentiation [143]. However, these findings remain limited to preclinical models. Although mTORC1 is a major nutrient- and amino acid-sensing pathway, its chronic hyperactivation may impair ISC function. In aged mice and conditional Tsc1-deletion models, sustained mTORC1 activation reduced ISC abundance and proliferative activity, disrupted villus and crypt architecture, and impaired post-irradiation regeneration through the MKK6–p38 MAPK–p53 stress pathway [72,147]. However, as mTORC1 activation stems from aging or a genetic Tsc1 deletion rather than dietary amino acid intervention, these findings provide only indirect evidence for amino acid-mediated ISC regulation.

Preclinical evidence suggests that glutamine may support ISC-mediated epithelial development and repair through context-dependent mechanisms. In early-weaned mice and murine enteroids, alanyl-glutamine supplementation enhanced ISC activity and epithelial development in association with WNT activation [148]. In a burn-injury mouse model supported by organoid and IEC-6 cell experiments, glutamine promoted ISC proliferation by reducing TIGAR promoter methylation and facilitating TIGAR-dependent YAP nuclear translocation [149]. However, whether these effects extend to human ISCs remains unclear. Evidence from poultry studies suggests that methionine and arginine may support intestinal barrier function through antioxidant, immunomodulatory, and metabolite-mediated mechanisms. However, because these studies did not directly assess Lgr5^+^ ISCs, they provide only indirect evidence for mammalian ISC regulation [150]. Using complementary porcine in vivo, ex vivo, and in vitro models, Dou et al. found that L-glutamate promoted ISC expansion and mitochondrial biogenesis in association with activation of the EGFR–MEK–ERK–TFB2M axis. These findings suggest that glutamate may function as both a metabolic substrate and a nutrient-responsive signal; however, their relevance to human ISC biology remains uncertain [151]. In aged Drosophila, asparagine supplementation limited ISC hyperproliferation and intestinal dysfunction by activating autophagy–lysosomal pathways and suppressing MAPK signaling [152].

However, given that the Drosophila intestinal stem cell differs substantially at the molecular level from the mammalian Lgr5^+^ crypt base columnar cell, such invertebrate findings should be regarded as conceptually suggestive rather than directly translatable to human ISC biology. Using murine irradiation-injury models, Chi et al. showed that a cysteine-rich diet enhanced Lgr5^+^ ISC-mediated regeneration through an epithelial–immune circuit. Epithelial cysteine uptake and CoA biosynthesis promoted the expansion of intraepithelial CD8αβ^+^ T cells and IL-22 production, thereby increasing ISC reparative capacity [153]. This response depended on epithelial SLC7A11, CD8αβ^+^ T cells, and IL-22, indicating that cysteine acts through immune-niche crosstalk rather than a solely ISC-intrinsic mechanism. However, its relevance to humans remains uncertain.

Collectively, amino acid effects on ISC biology are highly context-dependent and vary according to amino acid identity, dose, injury status, and experimental model. Because most evidence remains preclinical, the translational relevance and long-term safety of amino acid-based interventions remain uncertain. Enhanced ISC proliferation should therefore not be assumed to be beneficial in all physiological or pathological settings.

### 5.3. Lipids

Lipid-related regulation of ISC biology involves distinct processes, including endogenous fatty acid synthesis, lipid-derived signaling, receptor-mediated nutrient sensing, and cholesterol homeostasis. However, direct evidence on the effects of dietary lipid intake and fatty acid composition remains more limited than evidence from genetically or pharmacologically manipulated experimental models [21,95,147,154]. Using mice with intestinal epithelial-specific Acc1 deletion and intestinal organoid models, Li et al. [155] found that impaired de novo fatty acid synthesis reduced Lgr5^+^ ISC abundance, disrupted crypt architecture, and impaired organoid growth and self-renewal, alongside reduced nuclear accumulation of PPARδ and β-catenin. Although palmitate supplementation in organoids and a short-term high-fat diet in Acc1-deficient mice partly mitigated the observed defects, the effects of different dietary fatty acids and the relevance of these findings to human ISC biology remain unclear [155].

Lipid-dependent regulation of intestinal regeneration also involves peroxisomal remodeling. Using mouse colitis and Drosophila intestinal injury models, Guo et al. found that injury-induced release of very-long-chain fatty acids regulated ISC peroxisome abundance through a PPAR–SOX21 negative-feedback loop. Although this finding extends the role of lipids beyond energy provision, its relevance to human ISC biology remains unclear owing to the absence of human validation [156]. In genetic mouse models, MG53 increased endogenous unsaturated fatty acids and PPARα activity, promoting asymmetric ISC division toward secretory progenitor lineages. Notably, epithelial repair improved despite a reduced proportion of Lgr5^+^ ISCs, suggesting that regeneration does not necessarily require expansion of the stem-cell pool. However, this mechanism reflects endogenous lipid signaling rather than a direct dietary fat intervention. Collectively, these studies indicate that lipid metabolism may regulate peroxisome dynamics and lineage commitment through distinct mechanisms and that regenerative outcomes cannot be inferred solely from ISC abundance or proliferation [157].

Lipid-derived bioactive molecules provide an additional layer of ISC regulation. Ceramides, lysophosphatidic acid (LPA), and prostaglandin E_2_ (PGE_2_) have been implicated in ISC proliferation, regeneration, and cell-fate regulation [21]. Using human intestinal adenoma and tumor tissues together with mouse intestinal progenitor cells, organoids, and Drosophila models, Li et al. found that ceramide production increased PPARα-dependent FABP1 expression and fatty acid utilization, thereby promoting intestinal progenitor proliferation, stemness, and tumor potential in susceptible experimental models [158]. In murine irradiation-injury models supported by intestinal organoid experiments, epithelial-specific Lpar5 deletion suggested that LPA_5_ signaling supports ISC and progenitor survival and epithelial regeneration, in association with preserved Paneth-cell abundance and YAP-related repair signaling [159]. These findings indicate that lipid signaling is mediator- and context-dependent: LPA_5_ may support survival and repair following epithelial injury, whereas ceramide-driven PPARα–FABP1 signaling may promote pathological stemness in tumor-prone settings.

PGE_2_ represents another lipid-derived mediator involved in ISC regulation and intestinal epithelial repair [21]. In mouse small-intestinal organoids and a 5-fluorouracil-induced mucositis model, oxytocin stimulated PGE_2_ production and activated EP4-dependent Hippo/YAP signaling, thereby supporting ISC self-renewal and epithelial regeneration [160]. Lipid sensing may also regulate ISC lineage allocation. Using human small-intestinal enteroids from normal-weight donors and individuals with obesity, Farhadipour et al. found that pharmacological FFAR4 activation promoted differentiation toward lipid-processing absorptive enterocytes, increased lipid and iron storage, and reduced lipid peroxidation and susceptibility to ferroptosis. These responses were not observed in enteroids from donors with obesity, suggesting altered FFAR4-mediated lineage regulation in this metabolic context. However, these ex vivo findings do not establish the in vivo effects of dietary fatty acids on human ISC fate [161].

Patient-derived colonoids provide a distinct inflammatory context. In colonoids from pediatric patients with active ulcerative colitis, increased lipid accumulation, fatty acid oxidation, and PPARα activity were associated with hypermetabolic dysfunction, chemokine secretion, and cellular stress during epithelial differentiation. Pharmacological PPARα inhibition attenuated lipid accumulation and metabolic stress [162]. Together, these studies suggest that lipid-sensitive epithelial responses are modified by host metabolic and inflammatory status. Nevertheless, direct comparison is limited by differences in intestinal region, donor phenotype, disease context, and pharmacological target, and neither study provides in vivo evidence regarding dietary fat exposure.

Evidence from human Crohn’s disease tissues, mouse colitis models, and conditional Snx10 deletion experiments indicates that intracellular cholesterol biosynthesis can influence ISC maintenance during mucosal injury. SNX10 inhibition or deletion enhanced SREBP2-dependent cholesterol synthesis, preserved the ISC compartment, and promoted mucosal repair [163]. However, these findings concern intracellular cholesterol metabolism in inflammatory models and should not be interpreted as evidence that dietary cholesterol enhances intestinal regeneration. Conversely, Drosophila and mouse models, together with Crohn’s disease-derived organoids, have linked peroxisomal dysfunction and excessive cellular cholesterol accumulation to impaired YAP signaling and epithelial barrier dysfunction [164]. These contrasting findings suggest that cholesterol effects depend on its intracellular handling and disease context rather than cholesterol abundance alone.

Collectively, lipid metabolism regulates ISC maintenance and fate through fatty acid synthesis, lipid signaling, peroxisome function, and cholesterol homeostasis. Depending on context, these pathways may support epithelial repair or promote metabolic stress and tumor-associated stemness. Because human evidence is largely limited to ex vivo models and diseased tissues, the effects of dietary fat quantity and composition on human ISC function remain uncertain.

## 6. Micronutrients in Stem Cell Differentiation and Survival

Intestinal stem cells exert a significant influence on intestinal physiology by regulating the differentiation and proliferation of the intestinal epithelium [6]. However, macro and micronutrients regulate ISCs [6,21].

### 6.1. Vitamins

It has been reported that certain vitamins have beneficial effects on the microbiota, such as increasing alpha diversity in the colon, promoting the production of short-chain fatty acids, enhancing the abundance of beneficial commensals, and improving barrier function [6,165,166,167]. Recent studies have examined the effects of vitamins A, D, and B on ISCs [168,169,170,171,172,173,174].

In the intestinal epithelium, vitamin A acts via a signaling pathway to influence stem cell architecture and promote enterocyte differentiation through its retinoic acid form. It has been reported to play a role in enterocyte differentiation and the suppression of regenerative programs via retinoic acid (RA)-RAR/retinoid X receptor (RXR) activation [168,169]. In weaned piglets, increased dietary vitamin A has been reported to increase villus height and crypt depth. In the jejunum, it also increases the expression of Lgr5+ ISCs. This suggests that Vitamin A influences the stem cell pool and epithelial renewal; however, further studies are needed to understand the underlying mechanisms [21,170].

The effects of vitamin D on ISCs vary with dose, route of administration, model, and stage of cell differentiation. It has been demonstrated that Vitamin D receptor expression in human colon epithelial cells also affects Lgr5^+^ ISCs. In colon organoids, the application of calcitriol, the active form of Vitamin D, increases the expression of genes associated with stem cell properties—such as Lgr5^+^, SPARC-related modular calcium-binding protein 2 (SMOC2), and LRIG1—while suppressing cell proliferation. Accordingly, these cells have been reported to retain stem cell-like properties while exhibiting a lower proliferation rate [6,21,172]. Additionally, in tumor-derived colon organoids, cellular differentiation is promoted while proliferation is partially suppressed [171,172].

B vitamins influence ISCs through energy metabolism, mitochondrial function, epigenetic programming, and the microbiota. Niacin and vitamin B12, in particular, stand out due to certain properties. In a mouse model, some subtypes of ISC organoids were found to be more sensitive to 600 µg/mL niacin, with growth suppressed [173]. It is proposed that niacin regulates intestinal homeostasis, reduces inflammation, and protects the ISC niche via the mTOR signaling pathway [6,174].

Overall, much of the current evidence regarding the effects of vitamins on ISC biology has been derived from animal and organoid studies that provide mechanistic insights. However, well-designed human intervention studies confirming these findings are quite limited. Furthermore, differences in the bioavailability of vitamins, the administered dose and formulation, and the route of administration among experimental models contribute to the heterogeneity of the reported effects and make it difficult to directly translate existing preclinical findings into clinical practice. For this reason, well-designed human studies with standardized intervention protocols are needed to establish the role of vitamins in ISC biology in a more clinically reliable manner.

### 6.2. Mineral and Trace Elements

Minerals play a multifaceted role in regulating the ISC niche and maintaining epithelial integrity, exerting these effects through signaling pathways and the microbiota. Minerals such as iron, zinc, selenium, and magnesium are particularly prominent in this regard [6,175,176,177,178].

Evidence from mouse models indicates that iron plays a crucial role in the proliferation of intestinal epithelium and in the maintenance and protection of the crypt-villus architecture. However, excessive iron loading leads to a decrease in the villus-to-crypt ratio, microvillus damage, and suppression of ISC proliferation, while simultaneously increasing ferroptosis and oxidative stress [6,175,176].

Zinc is another mineral that significantly affects the ISC niche. Evidence from both in vivo mouse models and ex vivo enteroid models has demonstrated that zinc L-aspartate administration increases stem cell activity and supports the integrity of the epithelial barrier via the Wnt/β-catenin signaling pathway. Additionally, zinc deficiency has been reported to negatively affect both mineral metabolism and ISC function by reducing microbiota diversity [6,177].

Selenium, on the other hand, contributes to intestinal epithelial regeneration, particularly through its effects on the microbiota [6,178]. Additionally, in mice model, selenomethionine synthesized by selenium-enriched *Bifidobacterium longum* is reported to increase the number of LGR5^+^ ISCs, proliferative cell populations, and absorptive cell subtypes under dextran sulfate sodium (DON) exposure conditions [6,179]. Furthermore, it has been shown that selenium, applied in free form or as nanoparticles, suppresses reactive oxygen species formation, inflammatory cytokine responses, and mast cell activation by increasing the expression of various selenoenzymes, especially glutathione peroxidase 4 (GPX4); and that through these mechanisms, it contributes to the maintenance of epithelial barrier integrity and villus-crypt structure [180,181].

Magnesium, although less studied than the other minerals in the context of intestinal stem cell biology, is essential for intestinal epithelial proliferation. Intestinal Mg^2+^ uptake is mediated largely by the transient receptor potential melastatin channels TRPM6 and TRPM7, which act as principal gatekeepers of divalent mineral absorption across the epithelial barrier [182]. As Mg^2+^ is required as a cofactor for ATP utilization and cellular Mg^2+^ content must be approximately doubled during each cell division, TRPM7-dependent Mg^2+^ influx is closely associated with epithelial proliferative capacity; in colonic epithelial models, increased Mg^2+^ entry enhances proliferation and migration. Pharmacological or genetic restriction of Mg^2+^ availability can suppress these responses and is rescued by Mg^2+^ supplementation [183]. However, current evidence derives largely from whole-epithelium, cell-line, and cancer models rather than from lineage-defined Lgr5^+^ ISCs. The direct contribution of magnesium to stem cell self-renewal and differentiation remains to be elucidated.

In conclusion, current preclinical evidence suggests that minerals may play important roles in regulating the ISC niche, maintaining epithelial homeostasis, and supporting intestinal regeneration. However, the effects of minerals on ISC biology are not unidirectional; they may vary depending on the type of mineral, physiological concentration, duration of exposure, and cellular binding. Indeed, while zinc and selenium have been reported to support epithelial regeneration and barrier integrity, it has been shown that excessive iron loading can negatively affect ISC functions by increasing oxidative stress and ferroptosis. However, the fact that these mechanisms are largely based on findings from cell culture, organoid, and animal models indicates that the relationship between mineral homeostasis and ISC function in humans has not yet been fully elucidated. For this reason, translational research and well-designed human studies—which evaluate the effects of mineral balance on ISC biology under various physiological and pathological conditions and employ standardized intervention protocols—are of critical importance for translating current mechanistic findings into clinical practice. A summary describing the mechanisms of action of micronutrients on intestinal stem cells is given in Table 2.

## 7. Dietary Phytochemicals and Long-Term Intestinal Stem Cell Programming

### 7.1. Intestinal Stem Cells as Nutrition-Responsive Units

The intestinal epithelium undergoes rapid turnover approximately every 3–5 days, sustained by Lgr5^+^ ISCs located at the crypt base. ISC self-renewal and lineage commitment are governed by tightly regulated gradients of Wnt/β-catenin, Notch, and BMP signaling within the niche microenvironment [184,185]. Beyond classical morphogen regulation, increasing evidence indicates that ISC behavior is metabolically sensitive and responsive to nutritional cues [3,4].

Nutrient availability influences ISC biology through interconnected regulatory axes, including canonical Wnt/β-catenin signaling, nutrient-sensing pathways (AMPK–SIRT1–mTOR), redox-sensitive transcriptional programs such as Nrf2, and chromatin-modifying enzymes that determine transcriptional accessibility [3,4]. Because metabolic intermediates (e.g., NAD^+^, acetyl-CoA) function as cofactors for epigenetic enzymes, dietary inputs may shape epithelial transcriptional responsiveness without inducing permanent genetic alteration [186].

Within this framework, dietary phytochemicals—including polyphenols and other plant-derived bioactive compounds—may influence ISC output through two principal mechanisms: (i) direct modulation of stemness-related signaling pathways and (ii) indirect stabilization of epithelial integrity and niche conditions [98,186,187,188,189,190,191,192,193]. Representative mechanistic evidence supporting these pathways is summarized in Table 3.

### 7.2. Direct Modulation of ISC Signaling Pathways

#### 7.2.1. Morin: Receptor-Level Activation of Canonical Wnt Signaling

Morin, a flavonoid derived from mulberry leaves, is among the most mechanistically explicit examples of dietary ISC targeting. Morin binds to the Frizzled7 (FZD7) receptor and activates canonical Wnt/β-catenin signaling. In a heat-stable enterotoxin b injury model, morin promoted nuclear β-catenin accumulation, expanded the Lgr5^+^ ISC population, restored crypt–villus architecture, and enhanced organoid growth ex vivo [187].

Given that Wnt/β-catenin signaling is a core driver of ISC self-renewal, receptor-level engagement supports the concept that selected phytochemicals can directly recalibrate stemness circuits rather than merely acting as general antioxidants.

#### 7.2.2. Glucomannan (*Aloe vera*): Expansion of the ISC Compartment via Wnt/β-Catenin

Glucomannan isolated from *Aloe vera* gel has been shown to activate Wnt/β-catenin signaling in vivo. Treatment increased nuclear β-catenin localization, expanded the Lgr5^+^ ISC pool, and accelerated epithelial regeneration. Importantly, pharmacological inhibition of Wnt signaling attenuated these effects, confirming pathway specificity [189].

These findings indicate that certain bioactive polysaccharides can modulate the size of the ISC compartment along defined stemness axes, supporting epithelial renewal via direct signaling.

#### 7.2.3. Curcumin: Context-Dependent Wnt Recalibration Under Inflammatory Stress

In DSS-induced colitis, curcumin alleviated disease severity while inhibiting aberrant Wnt/β-catenin signaling and restoring the balance of ISC differentiation. Rather than simply stimulating proliferation, curcumin appeared to normalize excessive pathway activation under inflammatory stress [188].

This context-dependent recalibration is particularly relevant for long-term programming, as sustained epithelial renewal requires controlled signaling amplitude and appropriate lineage allocation rather than unchecked proliferative drive.

### 7.3. Indirect Modulation: Redox Stabilization and Functional Preservation of ISC Output

Not all bioactive effects require direct receptor-level engagement of stemness pathways. Several phytochemicals preserve ISC function indirectly by limiting oxidative injury, reducing inflammatory burden, and stabilizing the epithelial microenvironment.

#### 7.3.1. Quercetin: Nrf2-Dependent Stabilization of Regenerative Capacity

In a 10 Gy abdominal irradiation model, quercetin reduced epithelial apoptosis and DNA damage while enhancing regenerative proliferation in crypt regions via Nrf2 activation. Although lineage tracing was not performed, improved crypt regeneration under severe oxidative stress suggests preservation of ISC functional output [190].

In a complementary aging model, quercetin suppressed age-associated ISC hyperproliferation in Drosophila by attenuating reactive oxygen species and modulating insulin signaling [191]. Together, these findings support a redox-dependent stabilization of ISC dynamics across acute and chronic stress conditions.

#### 7.3.2. EGCG: Epithelial Protection and Regenerative Support via Nurr1 Activation

Epigallocatechin-3-gallate (EGCG) promotes epithelial proliferation and barrier restoration following ischemia/reperfusion injury. In vivo (12.5–50 mg/kg i.p.) and in vitro (40 mM intestinal epithelial cell line-6), EGCG enhanced epithelial proliferation, improved tight junction protein expression, and strengthened barrier integrity. These effects were attenuated by nuclear receptor-related 1 protein (Nurr1) knockdown, indicating Nurr1-dependent regulation [194].

EGCG has also been reported to mitigate radiation-induced intestinal injury, with features associated with the microbiota [193]. Within the scope of this section, microbiota modulation is interpreted as a secondary amplifier of epithelial recovery rather than the primary driver of ISC modulation.

Collectively, the studies discussed above indicate that phytochemicals such as morin, curcumin, quercetin, and EGCG may regulate intestinal stem cell (ISC) function through multiple complementary molecular pathways. Nevertheless, several important limitations should be considered before translating these promising findings into nutritional recommendations. To date, most mechanistic evidence has been generated using isolated phytochemicals administered under controlled experimental conditions in preclinical models, including cell culture systems, intestinal organoids, and animal studies, whereas direct evidence from well-controlled human intervention studies remains limited, restricting the immediate clinical translation of these observations [184,185]. Furthermore, many dietary phytochemicals exhibit inherently poor bioavailability because of limited intestinal absorption, extensive host and gut microbial metabolism, and rapid biotransformation, all of which substantially influence their systemic availability and biological activity in vivo. Moreover, the concentrations frequently employed in mechanistic studies often exceed those realistically achievable through habitual dietary intake, making direct extrapolation of experimental findings to human nutrition challenging [195]. It should also be recognized that the physiological effects of isolated phytochemicals cannot be directly equated with those of phytochemical-rich foods, as the food matrix, chemical stability, and interactions with other dietary constituents markedly influence phytochemical bioaccessibility, metabolism, and overall biological efficacy [196]. Therefore, future studies should combine physiologically relevant dietary exposures with advanced experimental models and well-designed human intervention trials to determine whether the promising mechanistic effects of dietary phytochemicals can be translated into evidence-based nutritional strategies for maintaining intestinal homeostasis and promoting epithelial regeneration.

### 7.4. Epigenetic Modifiers from Diet: Metabolic–Chromatin Coupling and Sustained ISC Programming

Long-term stem cell programming implies persistence beyond transient pathway activation. Several dietary polyphenols exhibit epigenetic regulatory potential, including modulation of DNA methyltransferase (DNMT) and HDAC activity [186]. Such chromatin-level effects provide biological plausibility for sustained alterations in transcriptional responsiveness following repeated dietary exposure.

Resveratrol exemplifies metabolic–epigenetic coupling in intestinal epithelium. By activating SIRT1, a NAD^+^-dependent deacetylase, resveratrol protected intestinal epithelial cells against radiation-induced damage by promoting autophagy [192]. Given that SIRT1 integrates metabolic state with chromatin remodeling, sustained exposure to sirtuin-activating compounds may influence epithelial stress adaptation and renewal dynamics.

**Figure 7:**
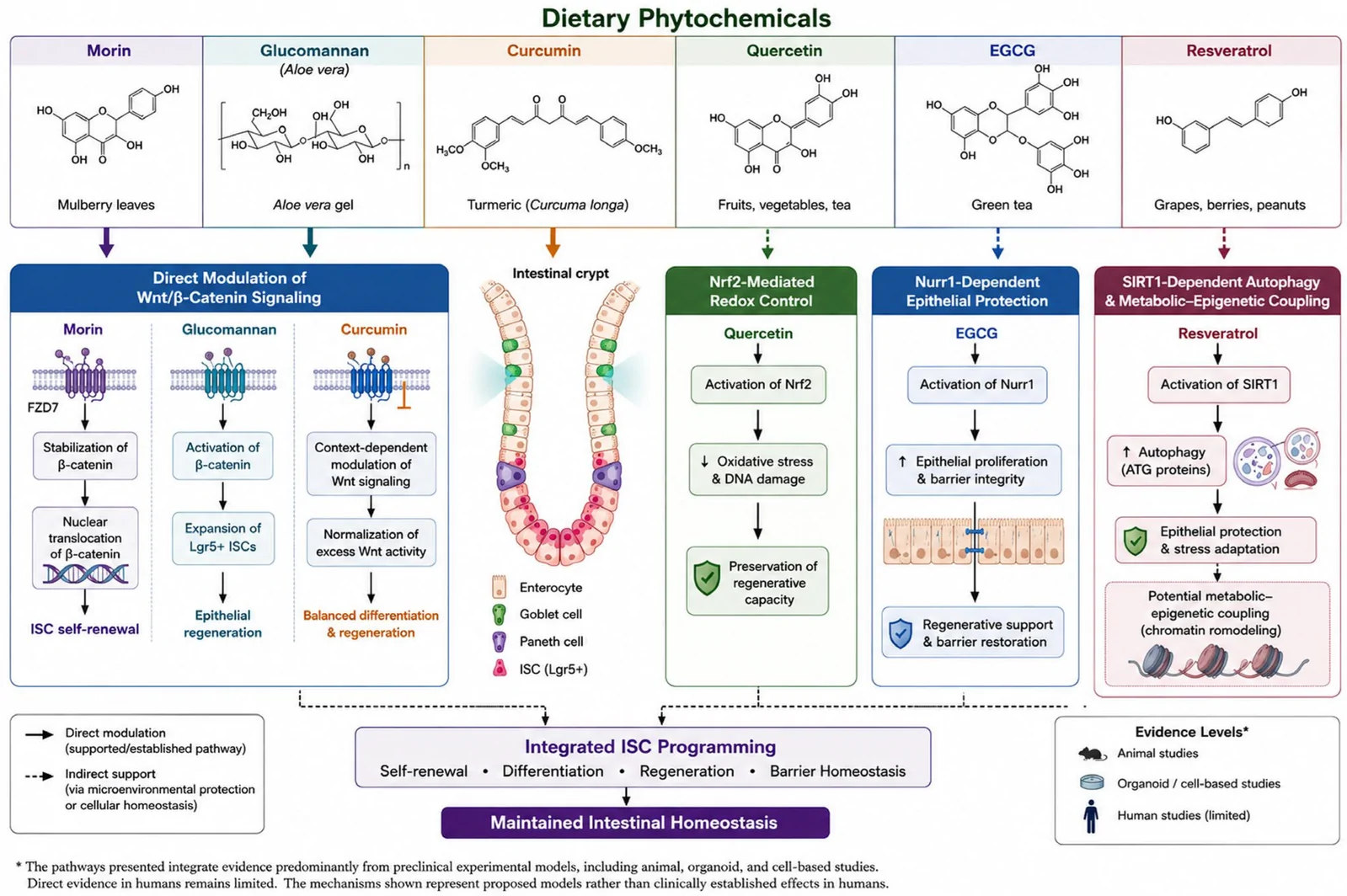
Dietary phytochemical–mediated regulation of ISC programming. Schematic representation of the proposed mechanisms through which dietary phytochemicals may influence intestinal stem cell (ISC) self-renewal, differentiation, and regenerative function. Selected phytochemicals act through direct or context-dependent modulation of Wnt/β-catenin signaling, whereas others provide indirect regenerative support through Nrf2-mediated redox control, Nurr1-dependent epithelial protection, SIRT1-mediated autophagy, and potential metabolic–epigenetic regulation. The depicted mechanisms integrate evidence predominantly from preclinical experimental models, including animal, organoid, and cell-based studies; direct evidence in humans remains limited. Accordingly, the pathways shown represent mechanistic frameworks rather than clinically established effects in humans. Abbreviations: ISC, intestinal stem cell; Wnt, wingless-type signaling pathway; Nrf2, nuclear factor erythroid 2-related factor 2; SIRT1, sirtuin-1; ATG, autophagy-related proteins.

Collectively, current mechanistic evidence indicates that dietary bioactives converge on a limited set of regulatory nodes—Wnt/β-catenin signaling, redox-responsive stabilization, and metabolic–epigenetic coupling—thereby shaping ISC output through both direct and indirect mechanisms. Although definitive lineage-traced epigenetic persistence within ISCs has yet to be demonstrated, these integrated signaling networks provide a plausible framework for sustained modulation of ISC behavior, as illustrated in Figure 7.

## 8. Diet–Microbiota–Stem Cell Axis

Diet plays a pivotal role in shaping the composition, function, and metabolic output of the intestinal microbiota [197]. The intricate interactions among diet, microbial communities, and their metabolites are essential for the establishment and maintenance of intestinal homeostasis [198]. The intestinal microbiota comprises a diverse array of microorganisms, including bacteria, fungi, archaea, and viruses [199]. The intestinal lumen is lined by a single-layered epithelium that is responsible for nutrient digestion and absorption, as well as protection against pathogenic invasion [200].

Stem cell proliferation and differentiation are regulated not only by intrinsic genetic mechanisms but also by nutritional status and metabolic cues. Factors such as caloric intake, dietary composition, and microbiota-derived metabolites significantly influence ISC function [201].

A healthy intestinal environment depends not only on stem cells but also on the surrounding microbiota and their metabolites, which directly influence stem cell behavior [199].

Emerging evidence highlights that microbiota-derived metabolites can modulate these regulatory networks and thereby influence epithelial renewal, regeneration, and tissue homeostasis [32]. However, the current body of evidence remains heterogeneous, encompassing findings derived from in vitro cell culture systems, intestinal organoids, animal models, and a comparatively limited number of human studies. Although these experimental models have substantially advanced the understanding of ISC biology, their findings cannot always be directly extrapolated to human physiology because of differences in microbial ecology, immune responses, and host metabolism. Therefore, careful interpretation is required when translating mechanistic observations into clinical applications [95].

Current evidence indicates that signals derived from both the host and the intestinal microbiota play a fundamental role in shaping the stem cell niche [199,202,203]. Disruptions in these regulatory mechanisms may impair stem cell function and increase susceptibility to diseases such as cancer and inflammatory bowel disease [204,205]. Intestinal microorganisms exert their effects through colonization and the production of bioactive metabolites, including short-chain fatty acids, succinate, indoles, and secondary bile acids, which regulate stem cell behavior [206]. For instance, studies using murine models demonstrated that metabolites derived from *Lactobacillus reuteri* enhance glycolysis and support ISC proliferation under stress conditions [207]. Conversely, evidence from experimental murine and intestinal organoid models suggests that microbiota-derived indole metabolites regulate ISC differentiation and epithelial homeostasis through aryl hydrocarbon receptor (AhR)-dependent signaling pathways [208]. Collectively, these findings support a regulatory role for microbiota-derived metabolites in ISC biology; however, the reported biological effects are not entirely consistent across studies. Variations in microbial composition, sequencing and metabolomic methodologies, experimental design, host genetics, dietary background, and environmental exposures may all contribute to these discrepancies. Furthermore, the relative contribution of individual microbial metabolites to ISC regulation remains incompletely understood, emphasizing the need for standardized experimental protocols and well-designed translational studies capable of validating these mechanisms in humans [95,209].

Microbiota-derived metabolites, including SCFAs, trimethylamine N-oxide (TMAO), and secondary bile acids, have been shown to influence stem cell proliferation and differentiation across different regions of the gastrointestinal tract, thereby affecting tissue integrity [210,211]. These metabolites can activate G protein-coupled receptors such as G protein-coupled receptor 41 (GPR41) and GPR43 [212]. Notably, GPR43 is expressed in intestinal epithelial cells and has been associated with protective effects against tumorigenesis by inhibiting excessive cell proliferation [213]. SCFAs play a crucial role in maintaining intestinal homeostasis by limiting excessive cell proliferation under physiological conditions [102]. Dysregulation of SCFA signaling, often associated with dysbiosis, may contribute to uncontrolled proliferation and increased cancer risk [214]. Nevertheless, the biological effects of SCFAs appear to be highly context-dependent. While most experimental studies report beneficial effects on epithelial integrity and ISC regulation, their biological activity is substantially influenced by SCFA concentration, intestinal microenvironment, microbial composition, host metabolic status, and disease stage. These factors likely explain the variability observed among experimental and clinical studies and illustrate the complexity of translating microbiota-derived metabolite research into clinical nutrition [202,215].

Another important class of metabolites, secondary bile acids, is produced through microbial transformation of primary bile acids synthesized in the liver from cholesterol [216]. While most bile acids are reabsorbed in the small intestine, a fraction reaches the colon, where they are converted into secondary bile acids such as deoxycholic acid and lithocholic acid by the gut microbiota [217].

Evidence derived predominantly from experimental animal models and mechanistic in vitro studies indicates that secondary bile acids exert complex and context-dependent effects on intestinal epithelial biology. Although physiological concentrations contribute to the maintenance of intestinal homeostasis by regulating host metabolism and cellular signaling, excessive accumulation under dysbiotic conditions has been associated with chronic inflammation, epithelial injury, DNA damage, and colorectal carcinogenesis. These apparently contradictory findings suggest that the biological effects of secondary bile acids are influenced by multiple factors, including microbial composition, bile acid concentration, exposure duration, and host metabolic status, rather than representing universally beneficial or detrimental molecules [218,219]. Consequently, further human studies are required to define the threshold at which alterations in bile acid metabolism become clinically relevant and to determine whether modulation of these pathways can be safely exploited for disease prevention or treatment. Interactions between diet and the intestinal microbiota play a central role in human health and disease [220]. These interactions influence systemic physiology not only through changes in microbial composition but also via microbial metabolites [2]. Compounds such as SCFAs, secondary bile acids, and tryptophan metabolites are critical regulators of energy metabolism, immune responses, inflammation, and even neurological functions [221]. The concentration and diversity of these metabolites are strongly influenced by dietary patterns, suggesting that diet-induced alterations in microbial metabolism may contribute to disease pathogenesis [199,222]. Despite substantial advances in this field, establishing direct causal relationships between dietary patterns, microbial metabolites, and ISC regulation in humans remains challenging. Most mechanistic insights have been generated under tightly controlled experimental conditions, whereas the human gut microbiota is shaped by numerous interacting factors, including genetics, age, medication use, lifestyle, geographical variation, and habitual dietary intake. This complexity limits the generalizability of pre-clinical findings and partly explains the variability observed across clinical studies. Therefore, future longitudinal human cohorts and randomized controlled dietary intervention studies integrating microbiome profiling, metabolomics, and functional analyses are essential to validate these mechanisms under real-world clinical conditions [202,223]. Dietary patterns can influence ISC function both directly and indirectly through alterations in microbial metabolism. Caloric restriction, high-fat diets, and ketogenic diets have all been reported to modify ISC activity and epithelial regeneration through changes in nutrient sensing pathways and metabolite availability. Emerging evidence suggests that a substantial proportion of these effects may be mediated through diet-induced alterations in the gut microbiota and its metabolic products [2,3,95,224]. However, much of the available evidence regarding these dietary patterns originates from experimental animal studies, whereas comparable human intervention studies remain relatively limited. Emerging evidence suggests that a substantial proportion of these effects may be mediated through diet-induced alterations in the gut microbiota and its metabolic products. Nevertheless, the magnitude and direction of these responses appear to vary considerably according to dietary adherence, intervention duration, baseline microbiota composition, metabolic health, and other host-specific characteristics. Such variability highlights the importance of considering inter-individual differences when interpreting current evidence and designing future nutritional interventions [95,209].

The majority of clinical studies have focused on the effects of dietary fiber, prebiotics, probiotics, whole grains, plant-based diets, and high-fat diets on the gut microbiota [199]. Evidence indicates that fiber-rich diets and prebiotic interventions promote the growth of beneficial bacteria such as Bifidobacterium and Lactobacillus, thereby enhancing microbial diversity and metabolic activity [225,226]. Dietary fibers act as primary substrates for beneficial gut bacteria, supporting their growth and function. Fiber-rich foods, including fruits, vegetables, legumes, and whole grains, provide a favorable environment for SCFA-producing microorganisms essential for intestinal health [227,228]. In contrast, diets high in fat and low in fiber are associated with dysbiosis and may lead to adverse metabolic outcomes [229]. Nevertheless, currently available dietary intervention studies differ substantially in dietary composition, intervention duration, participant characteristics, sequencing methodologies, and clinical outcome measures. This methodological heterogeneity complicates direct comparisons across studies and limits the formulation of universally applicable dietary recommendations [209,230]. Moreover, most available evidence remains associative rather than demonstrating direct causal relationships between diet-induced microbiota alterations and ISC regulation. Consequently, large-scale longitudinal cohorts and well-designed randomized controlled trials are required to establish causal mechanisms and identify the dietary strategies that provide the greatest clinical benefit [231]. Diet-based interventions that modulate the intestinal microbiota represent a promising strategy for disease prevention and management [232]. However, inter-individual variability in microbiota composition, dietary habits, and host genetics leads to heterogeneous responses to the same dietary interventions. This variability underscores the need for personalized approaches [233]. This biological variability underscores the limitations of a “one-size-fits-all” nutritional approach and supports the growing interest in precision nutrition. Accordingly, personalized nutrition strategies, including targeted use of prebiotics, probiotics, synbiotics, and microbiota-directed dietary models, are expected to play an increasingly important role in disease prevention and management [234].

Recent advances in microbiome profiling, metabolomics, metagenomics, and other multi-omics technologies have enabled the identification of microbial signatures and metabolite profiles that may eventually serve as biomarkers for individualized nutritional interventions. Integration of these biomarkers with dietary assessment, host genetic information, clinical characteristics, and environmental exposures may facilitate the development of precision nutrition strategies capable of preserving intestinal homeostasis, enhancing epithelial regeneration, reducing colorectal cancer risk, improving nutritional management of inflammatory bowel disease, and supporting healthy intestinal aging. Although these developments are highly promising, standardized biomarkers, validated prediction models, and clinically applicable decision-support tools remain limited. Consequently, additional translational research and prospective clinical studies are required before precision nutrition approaches targeting ISC biology can be routinely implemented in clinical practice [209,235,236].

In conclusion, growing evidence highlights the critical role of diet–microbiota interactions in regulating ISC function. A deeper understanding of how microbial metabolites influence stem cell homeostasis will facilitate the development of innovative, microbiota-based therapeutic strategies for disease prevention and treatment. From a translational perspective, accumulating evidence suggests that modulation of the gut microbiota through dietary interventions may contribute to future nutritional management of inflammatory bowel disease, colorectal cancer prevention, enhancement of intestinal regeneration following epithelial injury, and promotion of healthy intestinal aging. However, most mechanistic knowledge currently originates from animal models, intestinal organoids, and in vitro experimental systems, whereas robust evidence from well-controlled human intervention studies remains comparatively limited. Therefore, future research should prioritize longitudinal and multicenter clinical trials integrating microbiome profiling, metabolomics, functional microbiome analyses, and other multi-omics approaches to establish evidence-based precision nutrition strategies targeting ISC biology and to facilitate their translation into routine clinical practice.

## 9. Conclusions and Future Perspectives

**Figure 8:**
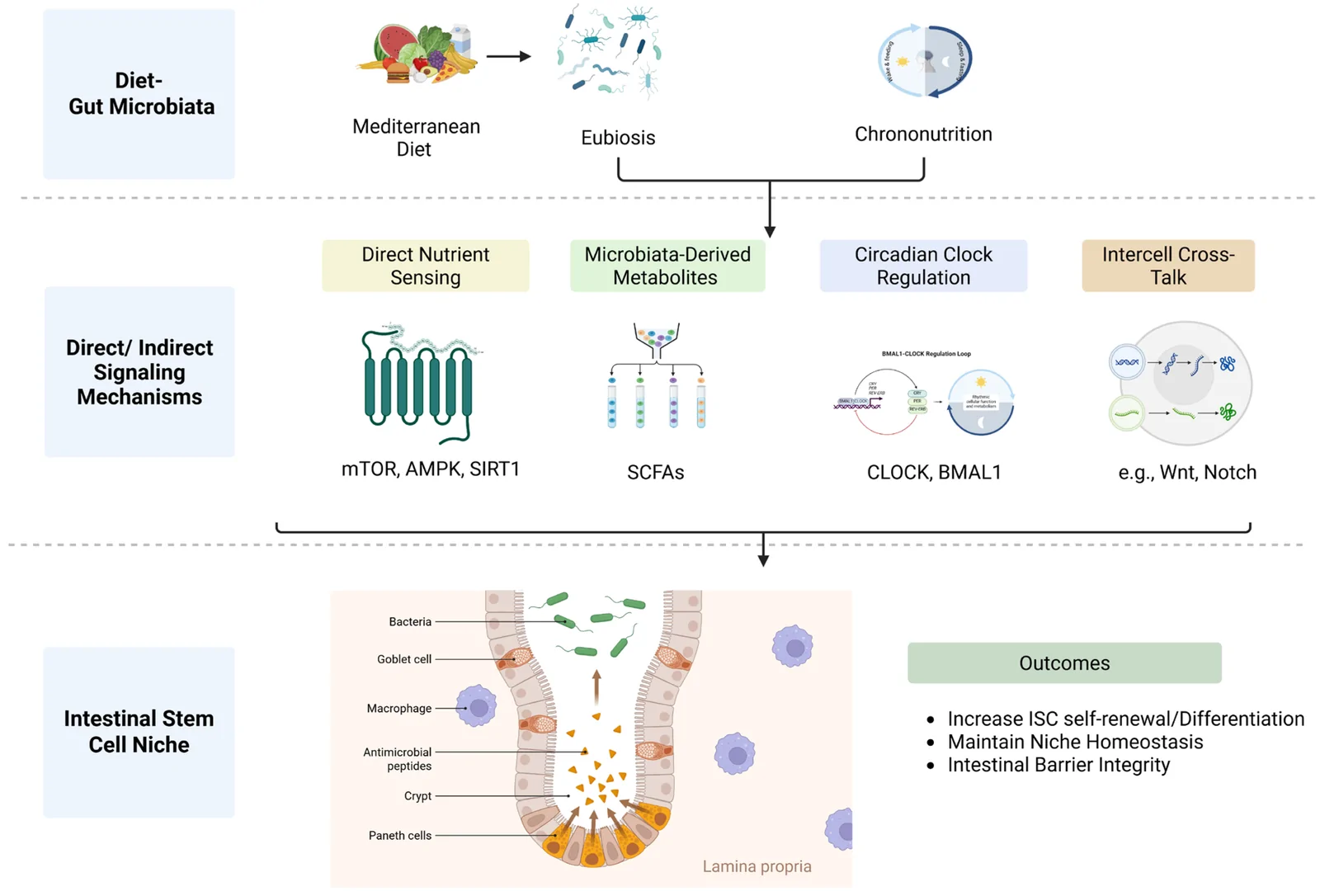
An integrated model of dietary regulation of intestinal stem cell behavior. Abbreviations: AMPK, AMP-activated protein kinase; BMAL1, brain and muscle ARNT-like 1; CLOCK, circadian locomotor output cycles kaput; ISC, intestinal stem cell; mTOR, mechanistic target of rapamycin; SCFAs, short-chain fatty acids; SIRT1, sirtuin 1; Wnt, wingless-type signaling pathway. Created in BioRender. Tok, B. (2026) https://BioRender.com/mmncxie, accessed on 13 August 2026.

Collectively, the current body of evidence suggests that intrinsic genetic programs do not solely govern ISC behavior but are dynamically regulated by a complex, multi-layered network of mechanisms influenced by dietary inputs. The effects of diet, gut microbiota, and direct and indirect signaling mechanisms on ISC behavior are summarized in Figure 8. Dietary patterns, macronutrients, micronutrients, and bioactive compounds appear to modulate ISC function both directly—via metabolic reprogramming and nutrient-sensing pathways such as mTOR, AMPK, and sirtuin signaling—and indirectly through alterations in the gut microbiota and its metabolite profile. This integrated framework, referred to in the literature as the “diet–microbiota–ISC axis,” is increasingly recognized as a critical regulatory system governing intestinal homeostasis and regenerative capacity.

The regulation of ISC activity through nutrient-sensing pathways—such as mTOR, AMPK, SIRT1, and PPAR signaling—as well as the influence of microbiota-derived metabolites on epithelial regeneration, has been extensively demonstrated in animal and organoid models. However, it remains unclear whether these mechanisms function analogously in the human intestine, whether dietary interventions can induce lasting epigenetic reprogramming of ISCs, and whether microbiota-targeted dietary strategies can causally modify human ISC function. These questions remain unresolved and should be considered as hypotheses requiring direct experimental validation rather than established facts. The effects of dietary interventions on ISC biology are highly context-dependent. While energy-restrictive strategies, including CR and fasting, have been shown to support stem cell function and enhance tissue regeneration, excessive nutrient intake—particularly within Western dietary patterns—has been associated with aberrant proliferation, metabolic dysregulation, and an increased risk of tumorigenesis. In addition, individual nutrients and metabolites may exert context-specific and sometimes dual effects depending on physiological conditions, niche-derived signals, and host-related factors, further highlighting the complexity of diet–stem cell interactions.

Despite considerable advances, important gaps remain in the literature. The majority of current mechanistic evidence is derived from animal models and in vitro organoid systems, which may not fully recapitulate human intestinal physiology. Direct evidence linking dietary interventions to functional changes in human ISC populations remains limited. Moreover, inter-individual variability in microbiota composition, genetic background, and metabolic status introduces additional layers of heterogeneity, complicating the translation of experimental findings into clinical practice.

Future research should prioritize integrating advanced methodologies, including single-cell multi-omics, spatial transcriptomics, and metabolomics, to better characterize ISC heterogeneity and niche interactions in response to dietary stimuli. Advanced human-derived organoid and organoid-on-chip models, combined with multi-omics and precision nutrition approaches, may help validate mechanistic findings, establish causal relationships, and identify interindividual variation in diet–ISC responses. Well-designed clinical studies will remain essential for determining whether these mechanistic insights can be translated into safe and effective individualized nutritional strategies targeting ISC biology. In addition, incorporating chrononutrition and feeding–fasting cycle dynamics into experimental frameworks may yield further mechanistic insight into the temporal regulation of ISC function.

From a translational perspective, a deeper understanding of diet-mediated regulation of ISC, including the roles of micronutrients, bioactive compounds, and microbiota-derived metabolites, may support the development of preventive and therapeutic strategies targeting intestinal disorders, age-related functional decline, and cancer risk. Ultimately, integrating stem cell biology with nutritional science represents a compelling and clinically relevant research direction, with the potential to inform the development of personalized, stem cell–targeted dietary interventions aimed at preventing intestinal pathology and restoring tissue homeostasis.

## Figures and Tables

**Table 1:** Summary of dietary interventions and their effects on intestinal stem cell biology across experimental models.

Dietary Intervention	Experimental Model	Main Effects on ISC Biology	Principal Mechanisms/ Pathways	Regenerative Outcomes	Tumorigenic Implications	Ref.
High-fructose diet	Mouse model	ISC-specific effects were not directly assessed; increased intestinal epithelial survival, villus length, and nutrient absorption without increased epithelial proliferation	Fructose metabolism via ketohexokinase; enhanced glycolytic intermediates and cellular survival	Improved epithelial cell survival; injury-induced regeneration not directly assessed	Increased epithelial survival may provide a growth advantage to neoplastic cells; ISC-specific tumor initiation not directly assessed	[96]
Western-style high-fat/high-sugar diet (HFHSD)	Mouse ISC reporter/lineage-tracing models; single-cell transcriptomics and in situ metabolomics	Increased Lgr5^+^ ISC and progenitor proliferation with accelerated differentiation and epithelial turnover, without expansion of the ISC pool	PPARγ/SREBP1-mediated lipogenesis and Insr–Igf1r–Akt signaling; reduced Wnt/β-catenin signaling	Accelerated epithelial turnover and lineage differentiation; injury-induced regeneration not directly assessed	Not directly assessed; chronic hyperproliferation and metabolic rewiring may contribute to a protumorigenic state	[101]
New Western Diet 1 (NWD1)	Mouse Lgr5^+^/Bmi1^+^ reporter and lineage-tracing models; single-cell transcriptomic and epigenomic analyses	Suppressed Lgr5^hi ISC function and lineage contribution with impaired progenitor maturation; compensatory recruitment of Bmi1^+^/Ascl2^hi alternative stem cells	Epigenetic Ppargc1a/PGC-1α suppression and impaired mitochondrial OXPHOS/TCA metabolism; Wnt–Ascl2-associated stem-cell plasticity	Compensatory stem-cell plasticity maintained epithelial homeostasis; injury-induced regeneration not directly assessed	Associated with a protumorigenic niche and altered tumor-initiating cell identity	[108]
Western diet (high-fat/high-sugar; ~40% fat)	Mouse models with epithelial/Paneth cell-specific FXR manipulation; ileal organoids; supportive human observational data	ISC-specific effects not directly assessed; induced Paneth cell dysfunction with potential consequences for the ISC niche	Microbiota/Clostridium–DCA–FXR and type I IFN signaling	Not directly assessed	Not assessed	[109]
High-fat diet (HFD)	Mouse model	Rapid increase in crypt proliferation followed by metabolic and lineage adaptation; enhanced absorptive enterocyte differentiation	Early stress-response programs followed by increased fatty-acid metabolism and lipid transport	Adaptive epithelial remodeling; injury-induced regeneration not directly assessed	Not directly assessed	[97]
High-fat diet (HFD)	Mouse models	Reduced MHC-II expression in Lgr5^+^ ISCs; MHC-II-deficient ISCs showed increased tumor-initiating capacity after Apc loss	Microbiota-associated suppression of epithelial MHC-II and impaired ISC–immune surveillance	Not directly assessed	Enhanced tumor initiation after Apc loss; ISC-specific MHC-II deficiency increased intestinal tumor burden	[99]
Ketogenic diet (KD)	Mouse models	Reduced Lgr5^+^ ISC proliferation and restored epithelial proliferative balance	β-Hydroxybutyrate–Hcar2–Hopx signaling	Not directly assessed	Suppressed colorectal tumor development in experimental models	[100]
Inulin-rich high-fiber diet	Mouse and germ-free models	Increased colonic epithelial proliferation and stemness with crypt remodeling; response was microbiota- and immune-niche-dependent	Microbiota–γδ T cell–IL-22 signaling; effects not solely dependent on FFAR2-mediated SCFA signaling	Enhanced epithelial remodeling; injury-induced regeneration not directly assessed	Long-term tumorigenic consequences not directly assessed	[110]
High-fiber diet	Mouse models	Increased colonic epithelial proliferation and stem-like properties, modulated by hypoxic signaling	HIF-1-dependent attenuation of high-fiber diet-induced proliferative/stemness responses	Supports context-dependent epithelial remodeling; injury-induced regeneration not directly assessed	Not directly assessed	[111]
Mediterranean diet intervention	Human randomized controlled trial	ISC function not directly assessed; microbiota-dependent changes in bile-acid metabolism may indirectly modify the intestinal metabolic environment	Gut microbiota–bile acid interactions	Not assessed	Not assessed	[98]
Mediterranean-like fat blend	Mucin-2-deficient mouse colitis model	ISC function not directly assessed; reduced intestinal inflammatory burden may indirectly improve the stem-cell niche	Modulation of diet-associated inflammatory and microbiota-related responses	Reduced colitis severity; ISC-mediated regeneration not directly assessed	Not assessed	[112]

Abbreviations: ISC, intestinal stem cell; HFD, high-fat diet; HFHSD, high-fat/high-sugar diet; KD, ketogenic diet; NWD1, New Western Diet 1; DCA, deoxycholic acid; FXR, farnesoid X receptor; IFN, interferon; OXPHOS, oxidative phosphorylation; TCA, tricarboxylic acid; SCFA, short-chain fatty acid; FFAR2, free fatty acid receptor 2; HIF-1, hypoxia-inducible factor 1.

**Table 2:** Micronutrients and their proposed mechanisms regulating intestinal stem cell biology.

Micronutrient	Experimental Model	Principal Mechanism/ Pathway	Effects on ISC Biology	**Translational Limitations**
**Vitamins**				
**Vitamin A** [21,170]	Weaned piglets	RA–RAR/RXR signaling	↑ Enterocyte differentiation, ↑ Lgr5^+^ ISC expression, epithelial renewal	No direct human ISC evidence
**Vitamin D** [6,21,171,172]	Human colon organoids, tumor organoids	Vitamin D receptor (VDR), calcitriol	↑ Lgr5, SMOC2, LRIG1 expression; ↓ proliferation; promotes differentiation	Dose-, model-, and stage-dependent effects
**Vitamin B (Niacin/B12)** [6,173,174]	Mouse ISC organoids	mTOR, mitochondrial metabolism, epigenetic regulation	Maintains ISC niche; niacin suppresses growth of sensitive ISC subtypes	Limited mechanistic and human data
**Mineral and Trace Elements**				
**Iron** [6,175,176]	Experimental mouse models studies	Oxidative stress, ferroptosis	Excess iron ↓ ISC proliferation, villus damage	Human intervention studies lacking
**Zinc** [6,177]	In vivo mouse models and ex vivo enteroid models	Wnt/β-catenin	↑ Stem-cell activity, epithelial barrier integrity	Human intervention studies lacking
**Selenium** [6,178,180,181]	DON mice model, selenium-enriched *B. longum*	GPX4, antioxidant defense, microbiota modulation	↑ Lgr5^+^ ISC number, regeneration, ↓ inflammation	Mostly animal evidence
**Magnesium** [182,183]	Cell lines, epithelial models	TRPM6/TRPM7, ATP metabolism	↑ Proliferation and migration; role in ISC remains unclear	No direct Lgr5^+^ ISC evidence

**Abbreviations:** *B. longum*, *Bifidobacterium longum*; DON, dextran sulfate sodium; GPX4, glutathione peroxidase 4; ISC, intestinal stem cell; Lgr5^+^, G protein-coupled receptor 5-positive cells; mTOR, mechanistic target of rapamycin; RA, retinoic acid; RAR, retinoic acid receptor; RXR, retinoid X receptor; SMOC2, SPARC-related modular calcium-binding protein 2; TRPM6, transient receptor potential melastatin 6; TRPM7, transient receptor potential melastatin 7; VDR, vitamin D receptor. ↑, increased; ↓, decreased.

**Table 3:** Representative experimental studies linking dietary phytochemicals to intestinal stem cell (ISC)–mediated epithelial regeneration and signaling regulation.

Dietary Phytochemical (Ref/Year)	Study Design	Model	Key Epithelial and ISC Outcomes	Primary Regulatory Axis	Modulation Type
Morin [187]	In vivo + ex vivo	STb-injury mouse model and intestinal organoids	Expansion of Lgr5^+^ ISC population; enhanced organoid growth; restoration of crypt–villus architecture	FZD7-mediated Wnt/β-catenin activation	Direct
Glucomannan [189]	In vivo	Mouse epithelial regeneration model	Expansion of the ISC compartment and accelerated epithelial regeneration	Wnt/β-catenin activation	Direct
Curcumin [188]	In vivo	DSS-induced colitis mouse model	Restoration of ISC differentiation balance and epithelial repair under inflammatory stress	Context-dependent modulation of Wnt/β-catenin signaling	Direct (context-dependent)
Quercetin [190]	In vivo	Abdominal irradiation mouse model	Reduced epithelial apoptosis and enhanced crypt regenerative capacity	Nrf2-mediated antioxidant signaling	Indirect
Quercetin [191]	In vivo	Drosophila aging model	Suppression of age-associated ISC hyperproliferation and improved epithelial homeostasis	ROS reduction and insulin signaling modulation	Indirect
EGCG [194]	In vivo + in vitro	Rat ischemia/reperfusion model and IEC-6 hypoxia/reoxygenation	Increased epithelial proliferation and improved tight junction integrity (ZO-1, occludin)	Nurr1 activation	Indirect
EGCG [193]	In vivo	Radiation-induced intestinal injury model	Reduced intestinal injury and improved epithelial recovery	Microbiota modulation with epithelial protection	Mixed
Resveratrol [192]	In vivo/in vitro	Radiation-associated epithelial injury models	Increased epithelial stress tolerance and epithelial survival	SIRT1-mediated autophagy signaling	Indirect (metabolic–epigenetic)

ISC, intestinal stem cell; ROS, reactive oxygen species; DSS, dextran sulfate sodium; IEC-6, rat intestinal epithelial cell line; ZO-1, zonula occludens-1; STb, heat-stable enterotoxin b.

## Data Availability

Data sharing is not applicable to this article as no new datasets were generated or analyzed during the current study.

## Abbreviations

The following abbreviations are used in this manuscript:

ACC1	Acetyl-CoA carboxylase 1
AhR	Aryl hydrocarbon receptor
AKT	AKT serine/threonine kinase/protein kinase B
AMPK	AMP-activated protein kinase
ATOH1	Atonal bHLH transcription factor 1
ATG	Autophagy-related proteins
ATG7	Autophagy-related 7
ATP	Adenosine triphosphate
BHB	β-Hydroxybutyrate
BMAL1	Brain and muscle ARNT-like 1
BMP	Bone morphogenetic protein
Bmi1	B lymphoma Mo-MLV insertion region 1 homolog
cAMP	Cyclic adenosine monophosphate
CBC	Crypt base columnar
CD8αβ^+^ T cells	CD8 alpha-beta-positive T cells
CD81	Cluster of differentiation 81
CLOCK	Circadian locomotor output cycles kaput
CoA	Coenzyme A
CR	Caloric restriction
CRY	Cryptochrome circadian regulator
CTGF	Connective tissue growth factor
DLL1	Delta-like canonical Notch ligand 1
DLL4	Delta-like canonical Notch ligand 4
DNA	Deoxyribonucleic acid
DNMT	DNA methyltransferase
DON	Deoxynivalenol
DSS	Dextran sulfate sodium
EGCG	Epigallocatechin-3-gallate
EGF	Epidermal growth factor
EGFR	Epidermal growth factor receptor
ERK	Extracellular signal-regulated kinase
FABP	Fatty acid-binding protein
FAO	Fatty acid oxidation
FFAR2	Free fatty acid receptor 2
FFAR4	Free fatty acid receptor 4
Foxl1	Forkhead box L1
FZD7	Frizzled class receptor 7
FXR	Farnesoid X receptor
GPX4	Glutathione peroxidase 4
GPR	G protein-coupled receptor
GPR41	G protein-coupled receptor 41
GPR43	G protein-coupled receptor 43
Grem1	Gremlin 1
Hcar2	Hydroxycarboxylic acid receptor 2
HDAC	Histone deacetylase
HES1	Hairy and enhancer of split 1
HIF-1	Hypoxia-inducible factor 1
Hippo	Hippo signaling pathway
Hopx	HOP homeobox
IEC-6	Rat intestinal epithelial cell line
IF	Intermittent fasting
IFN-γ	Interferon gamma
IL-22	Interleukin-22
I/R	Ischemia–reperfusion
ISC	Intestinal stem cell
ISCs	Intestinal stem cells
KD	Ketogenic diet
Lgr5	Leucine-rich repeat-containing G protein-coupled receptor 5
Lgr5^+^ CBC cells	Leucine-rich repeat-containing G protein-coupled receptor 5-positive crypt base columnar cells
Lgr5^+^/LGR5^+^	Leucine-rich repeat-containing G protein-coupled receptor 5-positive cells
Lkb1	Liver kinase B1
LPA	Lysophosphatidic acid
LPA5	Lysophosphatidic acid receptor 5
LRIG1	Leucine-rich repeats and immunoglobulin-like domains protein 1
MAPK	Mitogen-activated protein kinase
MEK	Mitogen-activated protein kinase/MAPK/ERK kinase
MHC	Major histocompatibility complex
MPC	Mitochondrial pyruvate carrier
mTert	Mouse telomerase reverse transcriptase
mTFB2	Mitochondrial transcription factor B2
mTOR	Mechanistic target of rapamycin
mTORC1	Mechanistic target of rapamycin complex 1
mTORC2	Mechanistic target of rapamycin complex 2
MYC	MYC proto-oncogene
NAD^+^	Nicotinamide adenine dinucleotide
Nrf2	Nuclear factor erythroid 2-related factor 2
Nurr1	Nuclear receptor related 1 protein
NWD1	New Western Diet 1
PDGFRα	Platelet-derived growth factor receptor alpha
PER	Period circadian regulator
PFKFB2	6-Phosphofructo-2-kinase/fructose-2,6-bisphosphatase 2
PGE2	Prostaglandin E2
PKA	Protein kinase A
PPARα	Peroxisome proliferator-activated receptor alpha
PPARδ	Peroxisome proliferator-activated receptor delta
PPARγ	Peroxisome proliferator-activated receptor gamma
PPARs	Peroxisome proliferator-activated receptors
PRRs	Pattern recognition receptors
PTGER4	Prostaglandin E receptor 4
RA	Retinoic acid
RAR	Retinoic acid receptor
ROS	Reactive oxygen species
RXR	Retinoid X receptor
SCFA	Short-chain fatty acid
SCFAs	Short-chain fatty acids
SIRT1	Sirtuin 1
SMOC2	SPARC-related modular calcium-binding protein 2
TA	Transient amplifying
TAZ	Transcriptional coactivator with PDZ-binding motif
TCA	Tricarboxylic acid cycle
TCF/LEF	T-cell factor/lymphoid enhancer-binding factor
TEAD	TEA domain transcription factor
TGF-α	Transforming growth factor alpha
TGR5	Takeda G protein-coupled receptor 5
TIGAR	TP53-induced glycolysis and apoptosis regulator
TMAO	Trimethylamine N-oxide
TRF	Time-restricted feeding
Tsc1/Tsc2	Tuberous sclerosis complex 1/tuberous sclerosis complex 2
Wnt	Wingless-type signaling pathway
Wnt/β-catenin	Wnt/β-catenin signaling pathway
YAP	Yes-associated protein
ZO-1	Zonula occludens-1
µg	Microgram
